# Under pressure: phenotypic divergence and convergence associated with microhabitat adaptations in Triatominae

**DOI:** 10.1186/s13071-021-04647-z

**Published:** 2021-04-08

**Authors:** Fernando Abad-Franch, Fernando A. Monteiro, Márcio G. Pavan, James S. Patterson, M. Dolores Bargues, M. Ángeles Zuriaga, Marcelo Aguilar, Charles B. Beard, Santiago Mas-Coma, Michael A. Miles

**Affiliations:** 1 Núcleo de Medicina Tropical, Faculdade de Medicina, Universidade de Brasília, Brasília, Brazil; 2 Faculty of Infectious and Tropical Diseases, London School of Hygiene and Tropical Medicine, London, UK; 3 Laboratório de Epidemiologia e Sistemática Molecular, Instituto Oswaldo Cruz-Fiocruz, Rio de Janeiro, Brazil; 4 Division of Vector-Borne Diseases, Centers for Disease Control and Prevention, Fort Collins, USA; 5 Laboratório de Mosquitos Transmissores de Hematozoários, Instituto Oswaldo Cruz-Fiocruz, Rio de Janeiro, Brazil; 6 Departamento de Parasitología, Facultad de Farmacia, Universidad de Valencia, Valencia, Spain; 7 Facultad de Ciencias Médicas, Universidad Central del Ecuador, Quito, Ecuador; 8 Instituto Juan César García, Quito, Ecuador

**Keywords:** Triatominae, *Rhodnius*, Chagas disease, Systematics, Morphometrics, Genetics

## Abstract

**Background:**

Triatomine bugs, the vectors of Chagas disease, associate with vertebrate hosts in highly diverse ecotopes. It has been proposed that occupation of new microhabitats may trigger selection for distinct phenotypic variants in these blood-sucking bugs. Although understanding phenotypic variation is key to the study of adaptive evolution and central to phenotype-based taxonomy, the drivers of phenotypic change and diversity in triatomines remain poorly understood.

**Methods/results:**

We combined a detailed phenotypic appraisal (including morphology and morphometrics) with mitochondrial *cytb* and nuclear ITS2 DNA sequence analyses to study *Rhodnius ecuadoriensis* populations from across the species’ range. We found three major, naked-eye phenotypic variants. Southern-Andean bugs primarily from vertebrate-nest microhabitats (Ecuador/Peru) are typical, light-colored, small bugs with short heads/wings. Northern-Andean bugs from wet-forest palms (Ecuador) are dark, large bugs with long heads/wings. Finally, northern-lowland bugs primarily from dry-forest palms (Ecuador) are light-colored and medium-sized. Wing and (size-free) head shapes are similar across Ecuadorian populations, regardless of habitat or phenotype, but distinct in Peruvian bugs. Bayesian phylogenetic and multispecies-coalescent DNA sequence analyses strongly suggest that Ecuadorian and Peruvian populations are two independently evolving lineages, with little within-lineage phylogeographic structuring or differentiation.

**Conclusions:**

We report sharp naked-eye phenotypic divergence of genetically similar Ecuadorian *R. ecuadoriensis* (nest-dwelling southern-Andean* vs* palm-dwelling northern bugs; and palm-dwelling Andean* vs* lowland), and sharp naked-eye phenotypic similarity of typical, yet genetically distinct, southern-Andean bugs primarily from vertebrate-nest (but not palm) microhabitats. This remarkable phenotypic diversity within a single nominal species likely stems from microhabitat adaptations possibly involving predator-driven selection (yielding substrate-matching camouflage coloration) and a shift from palm-crown to vertebrate-nest microhabitats (yielding smaller bodies and shorter and stouter heads). These findings shed new light on the origins of phenotypic diversity in triatomines, warn against excess reliance on phenotype-based triatomine-bug taxonomy, and confirm the Triatominae as an informative model system for the study of phenotypic change under ecological pressure.

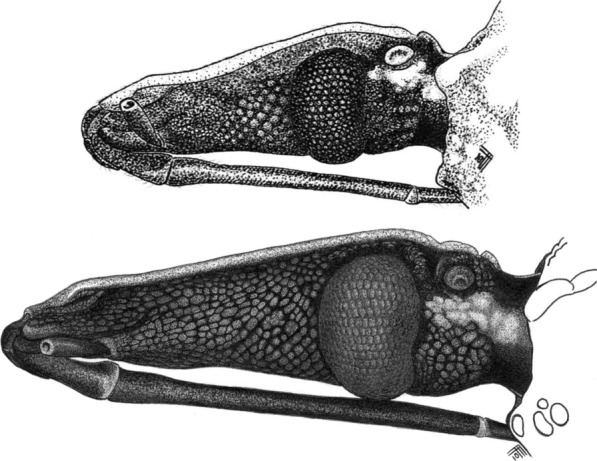

**Supplementary Information:**

The online version contains supplementary material available at 10.1186/s13071-021-04647-z.

## Introduction

Triatomine bugs transmit *Trypanosoma cruzi* among the mammalian hosts they associate with in shared microhabitats [1, 2]. Bugs that occur in human-made habitats may transmit the parasite to people, fueling the spread of Chagas disease [1, 3]. It has been proposed that occupation of new microhabitats may trigger selection for distinct phenotypic variants in these blood-sucking bugs [4]. Over the last few decades, molecular studies have identified examples of phenotypic convergence or divergence at several systematic levels [5–9]. Perhaps most remarkably, molecular phylogenetic analyses suggest that none of the three genera to which the main Chagas disease vectors belong (*Triatoma*, *Panstrongylus* and *Rhodnius*), which are all defined after morphological characters [1], is monophyletic [8–14]. A few named species have been shown to be phenotypic variants of another species [8]; for example, *Triatoma melanosoma* is now regarded as a *T. infestans* chromatic variant [15, 16]. Conversely, some named species have been shown to include several cryptic taxa [8]; for example, *Rhodnius robustus* (*s.l.*) is composed of at least five distinct lineages, two of which have been formally described as *R. montenegrensis* and *R. marabaensis* [8, 17–21]. Further examples of phenotype/genotype mismatch are reviewed in [8].

The sometimes striking variation of triatomine-bug phenotypes has been attributed to a propensity of morphological characters to change in response to changing habitat features [2, 4, 22]. Thus, within-species divergence may be driven by habitat shifts (e.g., wild to domestic) involving subsets of genetically homogeneous populations [4], and the use of similar habitats by genetically distinct populations may result in convergence or in the retention of ancestral phenotypes [2, 4, 23]. When using morphological characters only, therefore, taxonomists are in peril of describing spurious species or overlooking cryptic taxa [8]. Despite the practical importance of accurate taxonomic judgment when the organisms of interest transmit a life-threatening parasite, the degree, direction and underlying causes of phenotypic change and diversity in triatomines remain obscure.

**Fig. 1:**
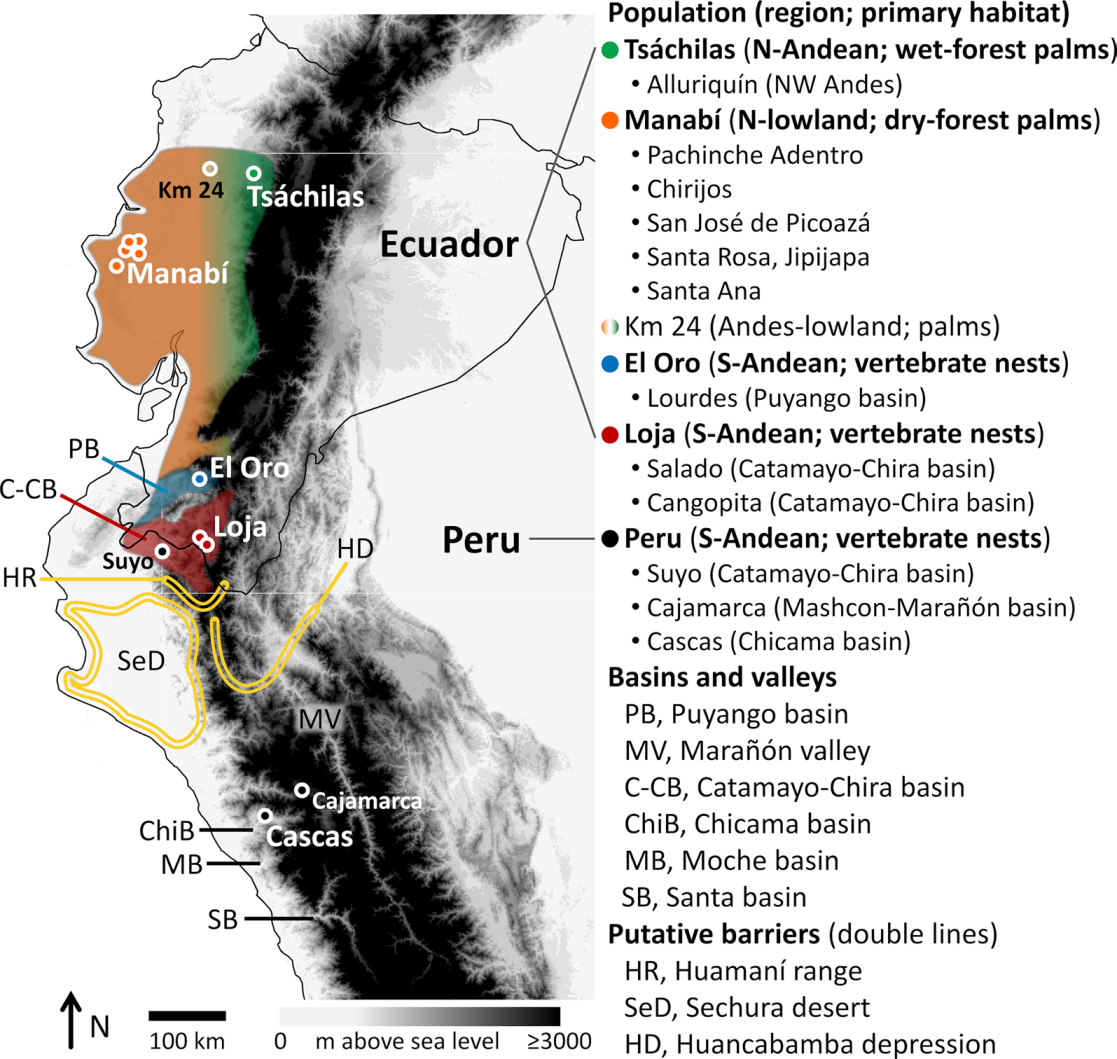
Sampling of *Rhodnius ecuadoriensis* populations. The map shows the approximate known distribution of *R. ecuadoriensis* in Ecuador, including the western-Andean wet premontane (or “cloud”) forests (green), the drier coastal lowlands (orange), the Andes–lowland transition and the southern, dry inter-Andean valleys associated with the Puyango (*PB*; blue) and Catamayo-Chira basins (*C-CB*; red). The approximate geographic location of each study population in Santo Domingo de los Tsáchilas, Manabí, El Oro, Loja and Peru is indicated by green, orange, blue, red and black circles, respectively; just one specimen was available from both Suyo and Cajamarca. Most Peruvian material came from Cascas, in the middle-upper Chicama basin (*ChiB*), La Libertad. Putative barriers to past or current bug dispersal are indicated with gold-colored double lines: the Huamaní range (*HR*), which closes off the C-CB to the south; the Sechura desert (*SeD*) on the northern Peruvian coastal plains; and the Huancabamba depression (*HD*). The semiarid Santa river basin (*SB*) appears to mark the southern limit of *R. ecuadoriensis*’ range. *Rhodnius ecuadoriensis* also occurs along the middle-upper (inter-Andean) stretches of the Marañón river valley (*MV*). See details in Additional file 1: Table S1 and main text

In this paper, we combine a detailed phenotypic characterization, qualitative and quantitative, with mitochondrial cytochrome* b* gene (*cytb*) and nuclear ribosomal second internal transcribed spacer (ITS2) DNA sequence analyses to study *Rhodnius ecuadoriensis* populations spanning most of the geographic/ecological range of the species (Fig. 1). *Rhodnius ecuadoriensis* is a major vector of *T. cruzi* in western Ecuador and northwestern Peru [24–26]. In Ecuador, northern wild populations are primarily associated with the endemic *Phytelephas aequatorialis* palm in both Andean wet forests and lowland dry forests; in the dry inter-Andean valleys of southwestern Ecuador, where native palms are rare or absent, wild *R. ecuadoriensis* seem to primarily associate with arboreal squirrel nests [1, 2, 27–33]. The natural habitats of Peruvian populations remain unclear, with a few records suggesting association with vertebrate nests/refuges in hollow trees and perhaps cacti [2, 26, 27, 34]. In addition, some *R. ecuadoriensis* populations have adapted to live in and around houses in coastal Ecuador and, especially, in the dry valleys of southwestern Ecuador and northwestern Peru—where the bugs contribute to endemic Chagas disease [24–26, 35–39]. In coastal and in southwestern Ecuador, bugs from wild and human-made habitats have overlapping phenotypes [40] and identical or nearly identical mitochondrial *cytb* haplotypes [41]; this, together with frequent, rapid reinfestation of insecticide-treated dwellings [36, 42] and preliminary microsatellite data [41, 43], indicates that wild and non-wild *R. ecuadoriensis* populations are locally highly interconnected. Our comparative phenotypic and genetic analyses cover most of the known geographic and ecological diversity in *R. ecuadoriensis*; they reveal that phenotypic divergence and convergence can both occur within a single nominal triatomine-bug species, and suggest that microhabitat adaptations likely play a crucial role in this phenomenon.

## Methods

### Origins of bugs and qualitative phenotype assessment

We compared *R. ecuadoriensis* type specimens from La Toma, Catamayo, Loja Province, Ecuador (Laboratório Nacional e Internacional de Referência em Taxonomia de Triatomíneos [LNIRTT], Fiocruz, Brazil; [44]) and canonical descriptions of the species [1, 45] with field-collected Ecuadorian bugs, including (i) northern bugs from *Ph. aequatorialis* palms of Santo Domingo de los Tsáchilas Province (Andean wet forests; “Tsáchilas” hereafter) and Manabí Province (lowland dry forests), and (ii) primarily nest-dwelling southern-Andean bugs caught in/around houses in El Oro (Puyango river basin) and Loja (Catamayo-Chira basin) provinces (Fig. 1). CAC Cuba (University of Brasília, Brazil) supplied additional field-caught southern-Andean Peruvian bugs from dwellings of Suyo (department of Piura, Catamayo-Chira basin) and Cascas (department of La Libertad, Chicama basin) [39] (Fig. 1). Finally, bugs from two colonies founded with material collected from, respectively, *Ph. aequatorialis* palms of Manabí and houses of northwestern Peru (department of Cajamarca, Mashcon-Marañón basin) were supplied by J Jurberg (LNIRTT) (see details in Additional file 1: Table S1). Using these specimens, which we note were fresh at the time of processing, we conducted a detailed review of external morphological and chromatic characters central to classical triatomine-bug taxonomy [1, 45] and placed the results in the broader context of what we know about the systematics, biogeography and ecology of *R. ecuadoriensis* [1, 2, 8, 18, 25–36, 38–43]. In particular, we emphasize that northern populations primarily exploit palm-crown microhabitats just like most *Rhodnius* species do [2, 29], whereas wild southern-Andean populations are primarily associated with vertebrate tree-nests in dry ecoregions where palms are either rare or absent [2, 8, 25–34]. Our sampling thus captures this key ecological difference, and we will hereafter refer to northern Tsáchilas and Manabí bugs as “primarily palm-dwelling” and to southern-Andean El Oro, Loja and Peru bugs as “primarily nest-dwelling” [2]. We note again that a growing body of evidence suggests that wild and non-wild *R. ecuadoriensis* populations are locally highly cohesive [36, 40–43].

### Traditional morphometrics—heads

**Fig. 2:**
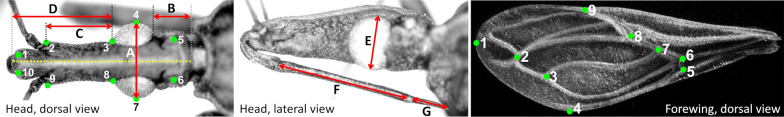
Measurements and landmarks used in the morphometric analyses. Head measurements were used for traditional morphometrics:* A* Maximum width across the eyes,* B* postocular distance (posterior eye limit to head/neck limit),* C* length of antenniferous tubercle (anterior eye limit to distal tip of tubercle),* D* anteocular distance (anterior eye limit to base of anteclypeus),* E* maximum diameter of the eye,* F* length of second rostral segment,* G* length of third rostral segment. The yellow dotted line indicates head length, which we used, together with* A*, to compute head length:width ratios. Green dots show the landmarks used for geometric morphometrics

We used 79 adult *R. ecuadoriensis* specimens for this part of the study; 77 were fresh bugs and two were collection bugs (see specimen details in Additional file 1: Table S1). We measured lateral- and dorsal-view, calibrated head images (Fig. 2) and calculated descriptive statistics for the whole sample and for ecological populations (primarily palm-dwelling *vs* primarily nest-dwelling) and geographic groups. To assess head-size variation, we estimated population means (over all head measurements) and likelihood-profile 95% confidence intervals (CIs) by fitting Gaussian generalized linear models (identity link-function, no intercept) using package *lme4* 1.1-21 [46] in R 3.6.3 [47]. For multivariate analyses, log-transformed data were centered by row to remove isometric size; the resulting “log-shape ratios” [48, 49] were used as input for principal component analysis (PCA) on covariances. The derived principal components (PCs or “shape variables”) were submitted to canonical variate analysis (CVA). We assessed the overall significance of multivariate CVA using Wilks’ *λ* statistic [50]. We computed canonical vectors (CVs) and used the first two CVs to plot the position of each specimen on the shape discriminant space; “convex hulls” enclosing all points within each group were overlaid on the plots. Finally, a space of size-free shape variables was constructed by explicitly removing size (represented by PC1) from the measurements; for this, residuals of linear regression of PC1 on each measurement were used as new variables for size-free CVA [51–53]. The derived CV1 and CV2 were plotted as described above. Different parts of these analyses were conducted using R 3.6.3 [47], JMP 9.0 (SAS Institute, Cary, NC, USA), and NTSYS 2.10y [54].

### Geometric morphometrics—heads and forewings

Dorsal-head and forewing images of, respectively, 84 and 82 adult bugs (all fresh except for 1 collection bug; see details in Additional file 1: Table S1) were digitized for a series of two-dimensional coordinates (Fig. 2) using *tpsDig* 1.18 [55]. Raw coordinates were subjected to the Procrustes superimposition algorithm [56] and thin plate spline (TPS) analysis using *tpsRelw* 1.18 [57]. We used TPS to compute “partial warps” with affine (global stretching) and non-affine (non-linear localized distortions or “shape changes”) components. PCA of partial warps yielded shape components (“relative warps”), which were subjected to CVA as described above. We also computed: (i) “centroid sizes” as overall measures of head and forewing size; and (ii) Mahalanobis distances between population pairs [22, 40, 49].

### Molecular analyses

We used 72 *R. ecuadoriensis* fresh specimens for mitochondrial DNA analyses and a subset of 17 bugs for nuclear DNA analyses (see details in Additional file 1: Table S1); *R. colombiensis*, *R. pallescens* and *R. pictipes* were used as outgroup taxa. We extracted DNA from bug legs using DNeasy kits (Qiagen, Valencia, CA). A 663-bp fragment of the mitochondrial cytochrome* b* gene (*cytb*) and the complete nuclear ribosomal ITS2 (707–715 bp) were amplified, purified and Sanger-sequenced as previously described [9, 11, 17]. We visually inspected the chromatograms of forward and reverse DNA strands with SeqMan Lasergene 7.0 (DNASTAR Inc., Madison, WI, USA); in inspecting ITS2 chromatograms, we particularly checked for the “double signal” typical of paralogous pseudogene sequences [58, 59]. We aligned consensus and outgroup sequences in MAFFT 7.0 [60], using the L-INS-i algorithm and further manual fine-tuning. We then computed descriptive statistics using MEGA X [61].

The best-fitting model of base substitution for each marker was selected using the Bayesian information criterion (BIC) in bModelTest 1.2 [62]. We used the *BEAST 0.15 package of the BEAST 2.6 platform [63, 64] to reconstruct Bayesian locus-specific phylogenetic trees and multispecies-coalescent species trees [63], with three independent runs (5 × 10^7^ generations) for each analysis. For locus-specific trees, we used the coalescent model and sampled parameters every 10,000 generations; for species trees, we used the Yule model of speciation and sampled parameters every 50,000 generations. We evaluated (using Tracer 1.7 [65]) parameter convergence and proper mixing by inspecting individual chains and by checking that effective sample sizes (ESSs) were sufficiently large; in our case, all ESSs were ≥ 10^4^. As a complement to phylogenetic analyses, we used *pegas* 0.14 [66] to build haplotype networks (infinite-sites model, Hamming distances) for both loci.

It has been suggested, based on limited mitochondrial DNA data, that *R. ecuadoriensis* may comprise two distinct lineages: one primarily from Ecuador (“group I”) and the other primarily from Peru (“group II”) [6, 18]. We set out to formally assess the data support for this “two-lineage” hypothesis (H_1_), relative to the null hypothesis of a single lineage (H_0_), by computing and comparing the marginal likelihood (mL) and posterior probability of each hypothesis [67, 68]. To do this, we first used our *cytb* and ITS2 sequence data to estimate species trees under both H_1_ (with each *R. ecuadoriensis* sequence assigned to a pre-defined, geography-based group, either “Ecuador” or “Peru”; Fig. 1) and H_0_ (with all *R. ecuadoriensis* sequences assigned to a single group). We then estimated the mL of each species tree using two approaches: (i) nested sampling [68, 69], as implemented in the NS 1.1 package [69] of BEAST 2.6 [63], with 5 × 10^6^ Markov chain Monte Carlo (MCMC) generations, 2 × 10^4^ sub-chain length and five active points; and (ii) path sampling (also known as “thermodynamic integration”) [68, 70], as implemented in the Model Selection 1.0.1 package of BEAST 2.6 [64], with a pre-burn-in of 2 × 10^5^ MCMC iterations followed by 90 steps of 2 × 10^6^ iterations (50% burn-in) and each step repeated 500 times. Using the hypothesis-specific log-mL values, we finally computed log-Bayes factors (BF) as log-BF = log-mL(H_1_) − log-mL(H_0_) [67]. For two hypotheses with equal prior probabilities, Pr(H_1_) = Pr(H_0_) = 0.5, the Bayesian posterior probability (BPP), given the data (*D*), of H_1_ is Pr(H_1_|*D*) = BF/(1 + BF), and the posterior probability of H_0_ is therefore Pr(H_0_|*D*) = 1 − Pr(H_1_|*D*). Kass and Raftery [67] proposed a set of rules of thumb, derived from those first suggested by Jeffreys [71], to grade the evidence in favor of H_1_: the evidence is weak when 1 ≤ BF < 3; positive when 3 ≤ BF < 20; strong when 20 ≤ BF < 150; and very strong when BF ≥ 150. In a two-hypotheses context like ours, this means that evidence in favor of H_1_ (or against H_0_) would be deemed very strong only if examination of the data changed the 1:1 prior odds (Pr(H_1_) = Pr(H_0_) = 0.5) to a posterior odds of at least 150:1, so that Pr(H_1_|*D*) ≥ 0.993 and Pr(H_0_|*D*) ≤ 0.007.

We note that the *R. ecuadoriensis cytb* sequences studied by Villacis et al. [41, 72] were not publicly available at the time of writing this report, when the National Center for Biotechnology Information (NCBI) GenBank held five such sequences. Sequence AF045715 [73] is only 399 bp, and sequences KC543508–KC543510 [74] are identical to some of the haplotypes we found (details below). Finally, KC543507 from a Manabí bug [74] differs at three positions from two of our Manabí haplotypes; one of those substitutions, however, yields a thymine at a second codon position (position 233 in Additional file 2: Alignment S1) where cytosine is conserved across all *cytb* sequences from *Rhodnius* species we have been able to examine (e.g., see [8]). This singular non-synonymous substitution (valine instead of alanine as in all other *Rhodnius* except *R. prolixus* and some *R. robustus*, which have threonine) suggests that KC543507 [74] may contain base-call errors, and we therefore excluded this particular sequence from further consideration. Our analyses, in sum, are based on all reliable *R. ecuadoriensis* sequences of reasonable length (here, 663 bp) that, as far as we know, are currently available for the two loci we investigated.

## Results

### Qualitative phenotypic assessment

**Fig. 3:**
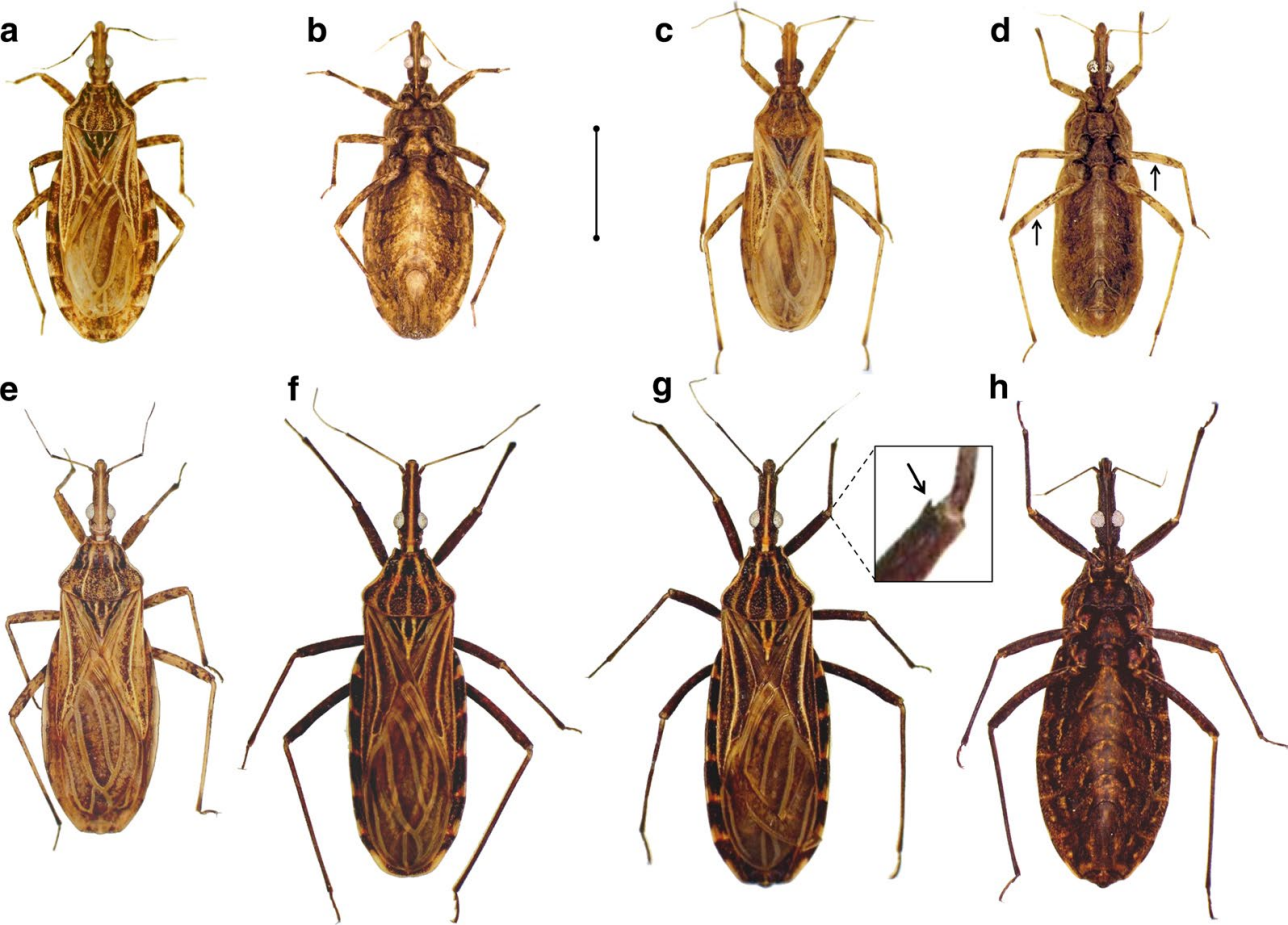
Phenotypic diversity in *Rhodnius ecuadoriensis*. **a**–**d** Southern-Andean populations (primarily from vertebrate tree-nests but often found in/around houses): Loja female, dorsal (**a**) and ventral (**b**) views; and Peru male, dorsal (**c**) and ventral (**d**) views; the arrows in **d** indicate the lighter central area of femora (see text). **e**–**h** Northern populations (primarily from *Phytelephas aequatorialis* palms): northern-lowland Manabí female, dorsal view (**e**), northern-Andean Tsáchilas male, dorsal view (**f**), northern-Andean Tsáchilas female, dorsal view (**g**; the inset highlights the well-developed denticle in the distal tip of the fore femora), northern-Andean Tsáchilas female, ventral view (**h**). Scale bar: approx. 5 mm

**Fig. 4:**
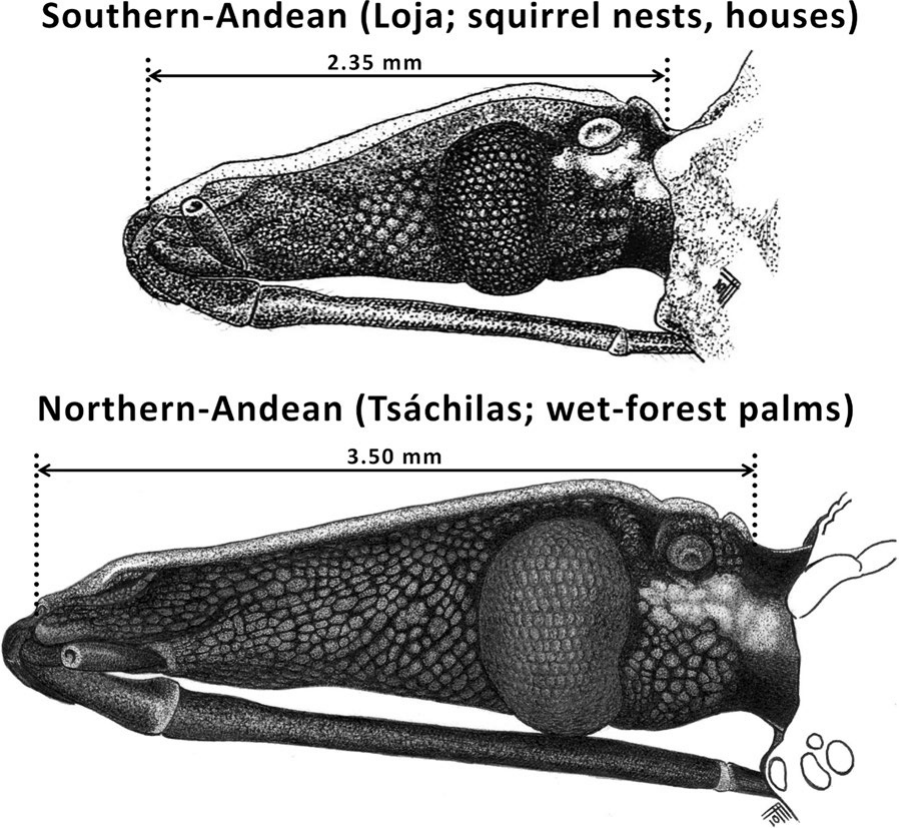
Heads (lateral view) of southern-Andean and northern-Andean *Rhodnius ecuadoriensis*: typical bugs primarily from vertebrate tree-nests (Loja)* vs* atypical bugs primarily from Andean *Phytelephas aequatorialis* palms (Tsáchilas). Note the striking divergence, which clearly falls within the range of what are normally considered interspecies differences in the Triatominae [1]. Drawings by FA-F

All southern-Andean, primarily nest-dwelling bugs from El Oro, Loja and Peru had comparable, typical phenotypes [1, 45], whereas northern-Andean specimens from wet-forest palms (Tsáchilas) were very large and dark bugs with long, slender heads; northern-lowland bugs from dry-forest palms (Manabí) had intermediate phenotypes (Figs. 3, 4). Below and in Table 1 we present summary descriptions of these diverse putative *R. ecuadoriensis* phenotypes (or “forms”); detailed descriptions are provided in Additional file 3: Text S1.

**Table 1:** Summary of phenotypic and ecological variation in *Rhodnius ecuadoriensis*

Form	Phenotypic traits^a^	Geography	Primary (wild) habitats^b^	Other habitats^b^
El Oro-Loja-Peru; typical phenotype^c^	Similar to species types; pale yellowish; mottled pattern conspicuous; small size (body length approx. 13–14 mm); short and stout heads; MPP pointed	Southern-Andean populations; inter-Andean valleys with dry forests in southwestern Ecuador (El Oro and Loja) and northwestern Peru (Pacific basins and middle-upper Marañón basin)	Squirrel tree-nests (Ecuadorian populations); hollow trees with *Didelphis* (Peruvian populations); likely also bird and rodent nests on trees and cacti	Houses and peridomestic structures; breeding colonies mainly associated with hen nests, dovecotes and guinea-pig pens
Manabí; intermediate phenotype	Light brown-yellowish; mottled pattern conspicuous; intermediate size (approx. 15–16 mm); heads longer than in typical specimens; MPP pointed	Northern-lowland populations; dry forests of coastal (i.e. western) Ecuador	*Phytelephas aequatorialis* palms	Squirrel, bird, rat or mouse nests; occasionally in man-made habitats (mainly peridomestic, with adult bugs often found invading houses)
Tsáchilas; highly atypical phenotype	Dark brown-black with brown-reddish markings; mottled pattern inconspicuous due to very dark background color; large (approx. 17–18 mm); long and narrow heads; MPP generally truncated	Northern-Andean populations; wet premontane (or “cloud”) forests (approx. 300–1800 m a.s.l.) along the Andes foothills in western Ecuador	*Phytelephas aequatorialis* palms	None known; adult (winged) bugs occasionally found invading houses

*MPP* Median process of the pygophore of the external male genitalia

^a^Detailed descriptions are provided in Additional file 3: Text S1

^b^See [1, 2, 24–43]

^c^Using the original description of the species [45] as the benchmark; see also [1]

#### Typical forms: primarily nest-dwelling southern-Andean bugs

Primarily nest-dwelling bugs caught in/around houses in southwestern Ecuador (El Oro, Puyango basin; and Loja, Catamayo-Chira basin) were virtually identical to the type material—very small triatomines, light brown-yellowish with dark-brown stripes and irregular markings on the body and appendages [1, 45] (Fig. 3). Peruvian bugs were collected in/around houses of the dry middle-upper Chicama basin (Cascas, La Libertad, approx. 350 km south of our fieldwork sites in Loja), except for one specimen collected in Suyo, Piura, within the Catamayo-Chira basin (Fig. 1; Additional file 1: Table S1). The overall aspect of Peruvian bugs largely matches that of type material (Fig. 3). However, Chicama-basin bugs are noticeably lighter-colored than southern-Andean Ecuadorian bugs; this is more evident on the legs, where the dark mottled pattern is limited to small clusters of dots and stripes on the basal and distal thirds of femora and tibiae (Fig. 3). The posterior lobe of the pronotum is also lighter than in Ecuadorian material, and Chicama-basin bugs are more slender than the typical specimens from El Oro-Loja. Similar to type material, the heads of these Peruvian specimens are noticeably short and stout (Fig. 3; see also Additional file 3: Text S1).

#### Intermediate forms: primarily palm-dwelling northern-lowland bugs

*Phytelephas aequatorialis* palms often harbor wild *R. ecuadoriensis* populations in the central coastal province of Manabí, Ecuador [2, 28, 29, 31]. The overall aspect and coloration of these lowland, primarily palm-dwelling bugs largely match those of typical *R. ecuadoriensis*, but Manabí bugs have larger bodies and longer, more slender heads (Fig. 3; see Additional file 3: Text S1).

#### Atypical forms: primarily palm-dwelling northern-Andean bugs

In 1998, a male *Rhodnius* specimen was collected at light near Alluriquín, Santo Domingo de los Tsáchilas, approximately 900 m a.s.l. on the central-western Ecuadorian Andes foothills (Fig. 1). This wet premontane forest site is within the range of *R. ecuadoriensis*, which is not shared by any other known *Rhodnius* species [8, 18, 25, 75], but the morphology and coloration of the specimen differed strikingly from those of *R. ecuadoriensis* type material (Table 1). Field surveys in Alluriquín yielded abundant material from *Ph. aequatorialis* palms [28, 76]. These bugs are much larger and darker, and have much longer heads, than typical *R. ecuadoriensis* (Table 1; Figs. 3, 4; Additional file 3: Text S1); they are, however, smaller than the closely related, light-colored *R. pallescens* and *R. colombiensis* [1, 77] (Additional file 4: Figure S1). Naked-eye phenotype differences between northern-Andean bugs and typical southern-Andean specimens are in the range customarily associated with distinct species in the Triatominae—and tend towards the “highly divergent” extreme of that range if we consider closely related species within the genus *Rhodnius* [1, 5, 8, 17–19] (Table 1; Figs. 3, 4; see also Additional file 3: Text S1 and Additional file 4: Figure S1).

### Traditional morphometrics—heads

**Fig. 5:**
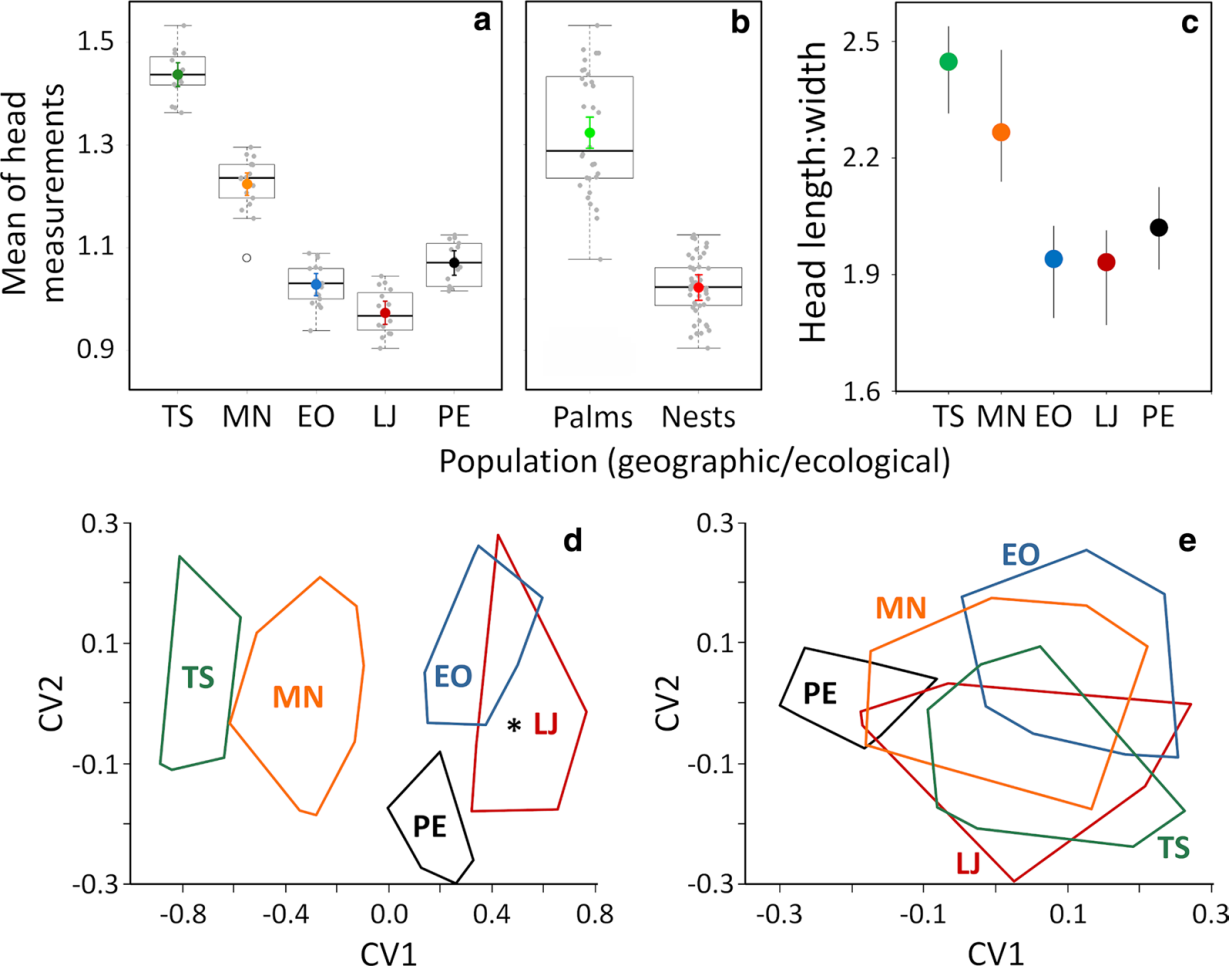
Traditional morphometrics of *Rhodnius ecuadoriensis* heads. Upper panels **a**–**c**: head size divergence among geographic (**a**) and ecological (**b**) populations. Population codes:* TS* Tsáchilas (dark green),* MN* Manabí (orange),* EO* El Oro (blue),* LJ* Loja (dark red),* PE* Peru (black). The primary habitat of each population is indicated in** b**: “Palms” for the the northern TS + MN populations (light green) and “Nests” for the southern-Andean EO + LJ + PE populations (bright red). Box plots (**a**,** b**) show medians (thick horizontal lines), quartiles (boxes) and values that fall within 1.5-fold the inter-quartile range (whiskers); note a single small-sized outlier (empty circle in **a**) in the MN population. Colored circles and error bars show population means and 95% confidence intervals. **c** Relation between head length (yellow dotted line in Fig. 2) and width (measurement “A” in Fig. 2), as the mean and range of raw length:width values; note the elongated heads of TS and MN bugs and the shorter, stouter heads of LJ, EO and PE bugs.** d**,** e** Size and shape divergence among geographic populations. **d** Isometry-free canonical discriminant analysis of linear head measurements; the asterisk indicates the position of a specimen from Suyo (Catamayo-Chira basin, Peru) in discriminant space. **e** Size-free canonical discriminant analysis on the residuals of linear regression of the first principal component on each head measurement.* CV* Canonical vector. See text for methodological details

Northern-Andean bugs from Tsáchilas palms clearly had the largest heads in our sample; northern-lowland bugs from Manabí palms were smaller than Tsáchilas specimens but larger than southern-Andean bugs—among which those from Loja had the smallest heads and those from Peru were larger on average (Fig. 5a). Overall, the heads of primarily palm-dwelling bugs were larger (Fig. 5b) and more elongated (Fig. 5c) than those of primarily nest-dwelling bugs. CVA confirmed among-group differences (Wilks’ *λ* = 0.024; *P* < 0.0001), with a negative correlation between CV1 scores and head size; again, discrimination between primarily palm-dwelling northern populations and primarily nest-dwelling southern-Andean populations was complete (Fig. 5d). Some overlapping occurred between El Oro and Loja, and a single Peruvian specimen was firmly nested within the Loja cluster (asterisk in Fig. 5d). This specimen was collected in Suyo, approximately 20–50 km from our fieldwork sites in Loja and also within the Catamayo-Chira basin (Fig. 1). When size effects were explicitly removed (see “Methods”), all Ecuadorian populations (plus the Suyo bug) were remarkably similar to one another, with bugs from Peru appearing as the most distinct (Fig. 5e).

### Geometric morphometrics—heads and forewings

**Fig. 6:**
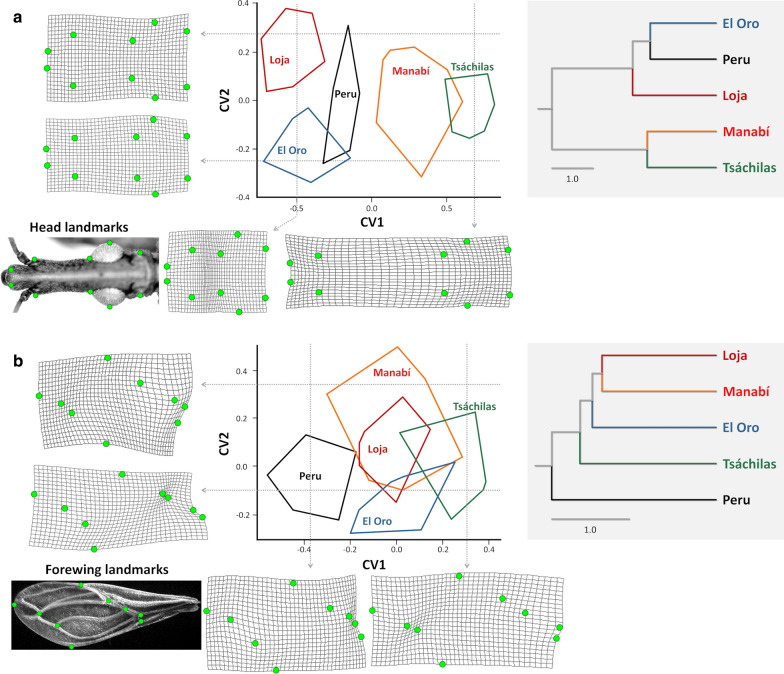
Geometric morphometrics of *Rhodnius ecuadoriensis* heads (**a**) and forewings (**b**): canonical discriminant analysis of relative warps. The left-hand-side plots are factorial maps based on the two first CVs, with convex hulls enclosing individual bugs of each population. Grids show head and forewing thin plate spline configurations at selected CV values (dotted arrows); head and forewing landmarks are shown (as light-green dots) for reference. Note the striking elongation of the head with increasing CV1 scores (in **a**) and the distinctive configuration of the forewing in Peruvian bugs, with low scores on both axes (in **b**). The right-hand-side panels show the results of UPGMA (unweighted pair group method with arithmetic means) cluster analyses based on Mahalanobis distances (scale bar: 1.0 unit). Centroid-size comparisons are shown in Additional file 5: Figure S2

Geometric head-shape analyses (Wilks’ *λ* = 0.051; *P* < 0.0001) confirmed the striking contrast between the large, elongated heads of northern, primarily palm-dwelling bugs (and, in particular, northern-Andean bugs from Tsáchilas) and the small, short-and-stout heads of primarily nest-dwelling southern-Andean bugs (Fig. 6a; see also Figs. 4, 5c). Centroid-size comparisons (Additional file 5: Figure S2) closely mirrored the results of the head-size analysis shown in Fig. 5a. The patterns revealed by CVA of forewing-shape components (Fig. 6b; Wilks’ *λ* = 0.016; *P* < 0.0001) were comparable to those revealed by size-free traditional head morphometrics (see Fig. 5e), although the divergence of Peruvian bugs was clearer against a backdrop of broad similarity among Ecuadorian populations (Fig. 6b). Thin plate splines and CV1 scores suggested that most Tsáchilas, some Manabí, and a few El Oro bugs had more elongated forewings, particularly in comparison with Peruvian material; most bugs from El Oro and Loja, as well as some Manabí specimens, were somewhat intermediate (Fig. 6b). Forewing centroid-size values were similar across primarily nest-dwelling southern-Andean populations, much larger in northern-Andean bugs, and again intermediate in northern-lowland Manabí bugs (Additional file 5: Figure S2).

### Molecular analyses 1—mitochondrial DNA

**Fig. 7:**
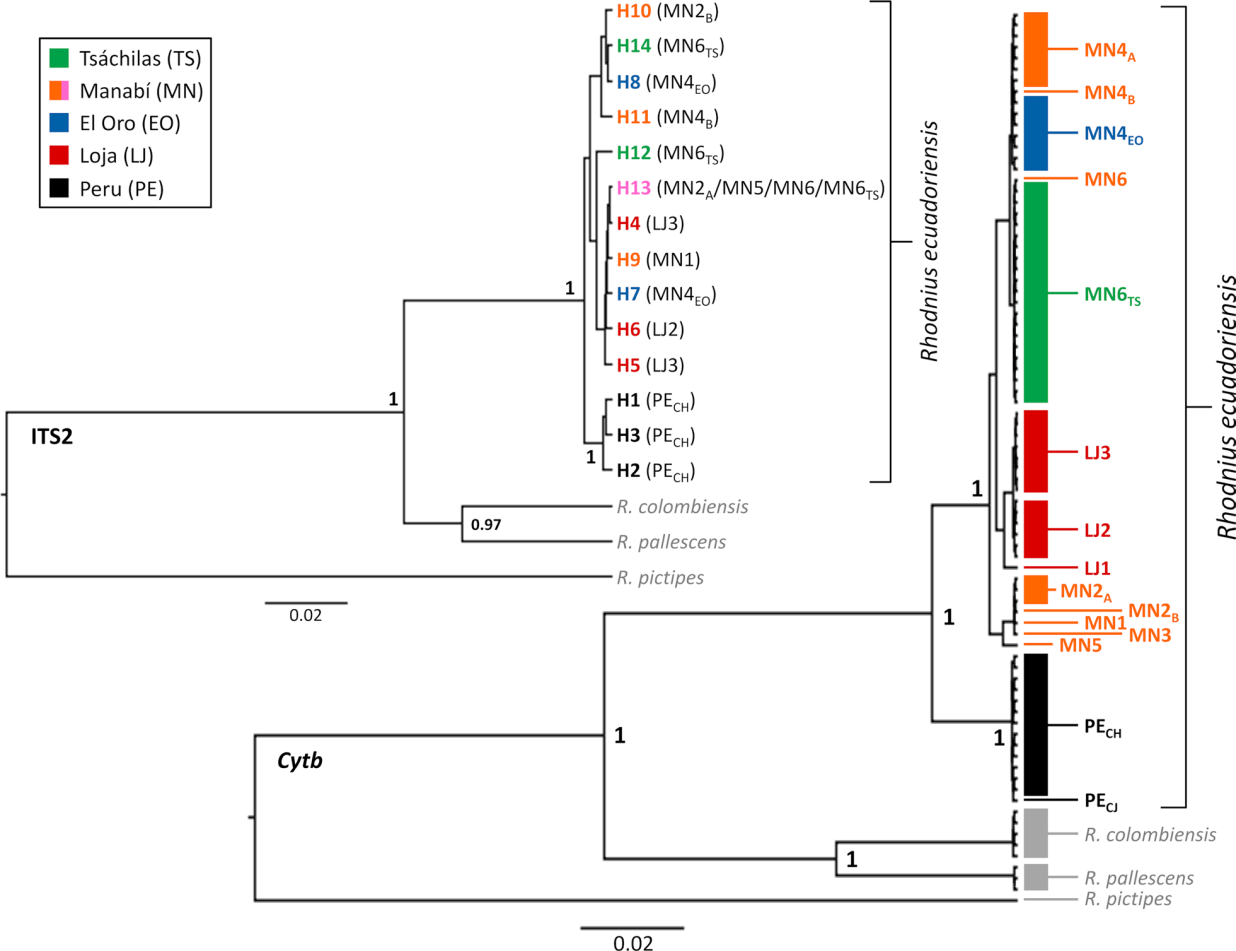
Phylogenetic relations among the mitochondrial cytochrome* b* gene (*cytb*) and nuclear ribosomal second internal transcribed spacer (ITS2) haplotypes in *Rhodnius ecuadoriensis*. Mitochondrial *cytb* haplotypes found in bugs with each ITS2 haplotype are shown in parentheses in the ITS2 tree. Note the lack of differentiation among Ecuadorian populations. Note also: (i) that ITS2 haplotype H13 (pink font) was found in several bugs from Manabí province and in one bug from Tsáchilas and (ii) that good-quality ITS2 sequences could not be determined for bugs with *cytb* haplotypes MN3 (Manabí, Ecuador) and PE_CJ_ (Cajamarca, Peru). Bayesian posterior probabilities > 0.95 are shown close to key nodes. Scale bars are in substitutions per site. Outgroup taxa are in gray font

**Fig. 8:**
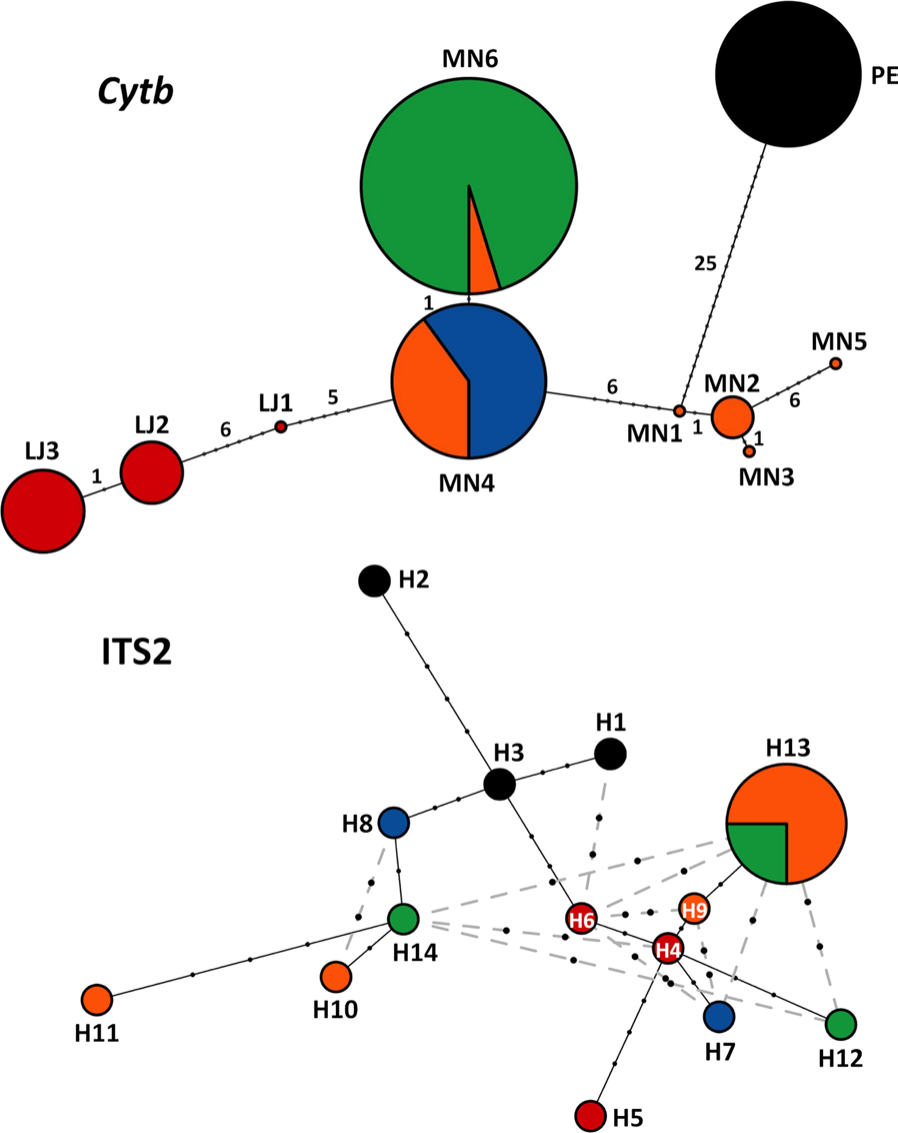
Networks of mitochondrial *cytb* and nuclear ITS2 haplotypes in *Rhodnius ecuadoriensis*. Each circle represents a haplotype, with circle size proportional to haplotype frequency. Mutational steps are represented by black dots, with numbers given for the smaller-sized dots of the *cytb* network. Alternative connections between haplotypes (represented by gray-broken edges) were only inferred for the ITS2 network. Color codes are as in Figs. 1, 5, 6, and 7

We found ten *cytb* haplotypes, some recovered from different collection sites, in our *R. ecuadoriensis* samples (see Additional file 1: Table S1; Figs. 7, 8; and below). All 663-bp sequences comprised an open reading frame with no stop codons or any other signs of pseudogene sequences. The *R. ecuadoriensis cytb* alignment had 42 variable sites (6.3%); 36 were in third codon positions, five in first codon positions, and one was in a second codon position. There were two non-synonymous point mutations in a single codon of the only haplotype (PE) found in all 13 Peruvian bugs—with ACT (threonine) instead of GTT (valine). One Manabí haplotype (MN5) also had a first codon position non-silent substitution—ATT (isoleucine) instead of CTT (leucine) (see Additional file 2: Alignment S1; sequences were deposited in GenBank under accession codes MT497021–MT497035 for *R. ecuadoriensis* and MT497036–MT497038 for outgroup taxa).

We isolated six *cytb* haplotypes (MN1–MN6) from northern-lowland Manabí bugs (Additional file 1: Table S1). MN1, MN3 and MN5 were found in one specimen each. MN2 was detected in bugs from two sites (codes MN2_A_ and MN2_B_); MN2 is identical to KC543509 from Santa Ana, Manabí [74]. MN4 was also found in bugs from two distinct Manabí sites (MN4_A_ and MN4_B_), as well as in all nine southern-Andean bugs from El Oro (MN4_EO_), including those collected in two different sites and in different years; MN4 is identical to KC543510, also from Santa Ana, Manabí [74]. MN6 was found in one bug from Manabí and in the 20 northern-Andean specimens from Tsáchilas palms (MN6_TS_). MN4 and MN6 differ by a single, third codon position C/T transition. We isolated three unique haplotypes (LJ1, LJ2 and LJ3) from the 15 southern-Andean Loja bugs (Additional file 1: Table S1); LJ2 is identical to KC543508 from Quilanga, Loja, in the Catamayo-Chira basin [74]. Finally, 13 Peruvian bugs from at least three dwellings of the middle-upper Chicama basin also yielded a single, unique haplotype (PE_CH_). One specimen from the reference *R. ecuadoriensis* colony at LNIRTT, founded in 1979 with bugs from Cajamarca, had the same haplotype (coded PE_CJ_) (Additional file 1: Table S1). Haplotype PE was the most distinct among all the *R. ecuadoriensis cytb* sequences we studied; it was separated by 25 nucleotide substitutions from the closest Ecuadorian haplotype (MN1; see Figs. 7, 8; Additional file 2: Alignment S1; and below).

Overall, uncorrected *cytb* nucleotide diversity was *π* = 0.0179 ± 0.0029 standard error (SE) (SEs estimated with 1000 bootstrap pseudo-replicates). Mean Kimura 2-parameter (K2p) distances were 0.0185 ± 0.0031 SE for the 72-sequence *cytb* alignment and 0.0184 ± 0.0029 SE for the ten-haplotype dataset. K2p sequence divergence was substantially lower among Ecuadorian haplotypes (0.0015–0.01995) than between any of these and PE (0.03904–0.04894). Mean K2p distance between the primarily palm-dwelling northern-Andean (Tsáchilas) and northern-lowland (Manabí) populations was low (0.0070 ± 0.0023 SE), and much lower than distances between these two populations and the primarily nest-dwelling southern-Andean bugs (0.0210 for Tsáchilas and 0.0222 for Manabí; both  ± 0.0037 SE). These larger distances were driven by the clearly divergent Peruvian haplotypes; when these were placed in a separate group, K2p distances between Ecuadorian populations were between 0.15 and 1.43%, whereas distances between Ecuadorian and Peruvian populations were all > 4.07% (Table 2).

**Table 2:** Kimura two-parameter distances between pairs of *Rhodnius ecuadoriensis* populations from across the species’ range

Marker	Population 1	Primary habitat	Population 2	Primary habitat	K2p distance	SE
*Cytb*	Tsáchilas (N)	Andean palms	Manabí (N)	Lowland palms	0.00697	0.00227
	Tsáchilas (N)	Andean palms	El Oro (S)	Vertebrate nests	0.00151	0.00149
	Tsáchilas (N)	Andean palms	Loja (S)	Vertebrate nests	0.01283	0.00419
	Manabí (N)	Lowland palms	El Oro (S)	Vertebrate nests	0.00566	0.00185
	Manabí (N)	Lowland palms	Loja (S)	Vertebrate nests	0.01426	0.00383
	El Oro (S)	Vertebrate nests	Loja (S)	Vertebrate nests	0.01129	0.00396
	Tsáchilas (N)	Andean palms	Peru (S)	Vertebrate nests	0.04235	0.00823
	Manabí (N)	Lowland palms	Peru (S)	Vertebrate nests	0.04127	0.00782
	El Oro (S)	Vertebrate nests	Peru (S)	Vertebrate nests	0.04072	0.00804
	Loja (S)	Vertebrate nests	Peru (S)	Vertebrate nests	0.04806	0.00868
ITS2	Tsáchilas (N)	Andean palms	Manabí (N)	Lowland palms	0.00308	0.00126
	Tsáchilas (N)	Andean palms	El Oro (S)	Vertebrate nests	0.00331	0.00148
	Tsáchilas (N)	Andean palms	Loja (S)	Vertebrate nests	0.00378	0.00152
	Manabí (N)	Lowland palms	El Oro (S)	Vertebrate nests	0.00402	0.00153
	Manabí (N)	Lowland palms	Loja (S)	Vertebrate nests	0.00402	0.00127
	El Oro (S)	Vertebrate nests	Loja (S)	Vertebrate nests	0.00473	0.00168
	Tsáchilas (N)	Andean palms	Peru (S)	Vertebrate nests	0.00519	0.00192
	Manabí (N)	Lowland palms	Peru (S)	Vertebrate nests	0.00638	0.00214
	El Oro (S)	Vertebrate nests	Peru (S)	Vertebrate nests	0.00614	0.00220
	Loja (S)	Vertebrate nests	Peru (S)	Vertebrate nests	0.00613	0.00221

Calculations are based on 72 mitochondrial cytochrome* b* (*cytb*) DNA sequences and 17 nuclear ribosomal second internal transcribed spacer (ITS2) DNA sequences from five populations primarily associated with two distinct microhabitats: *Phytelephas aequatorialis* palms to the north (N) and vertebrate tree-nests to the south (S); see Fig. 1, Table 1 and Additional file 1: Table S1.

*K2p* Kimura 2-parameter, *SE* Standard error computed from 1000 bootstrap pseudo-replicates

The best-fitting model for the *cytb* alignment had three substitution rates (AT = CG = GT; AG = CT; and AC) with a Gamma shape parameter (+*Γ*) and a proportion of invariable sites (+*I*). The *cytb* gene tree shows an unambiguous separation (BPP = 1.0) of the Peruvian haplotype from a monophyletic (BPP = 1.0) Ecuadorian clade. No clear patterns of geographic or ecological segregation are apparent within the Ecuadorian clade, although the three LJ haplotypes unique to Loja bugs (see Fig. 7) cluster together with BPP ≈ 0.95. These patterns are also apparent in the *cytb* haplotype network shown in Fig. 8.

### Molecular analyses 2—nuclear DNA

We identified 14 unique ITS2 haplotypes (GenBank codes KT267937–KT267950) in our *R. ecuadoriensis* sample, including three (H1–H3) from Peruvian bugs carrying the PE_CH_ *cytb* haplotype and 11 from Ecuadorian bugs: two from northern-Andean bugs (Tsáchilas, H12 and H14); three from northern-lowland Manabí bugs (H9–H11); one (H13) from several Tsáchilas and Manabí bugs; two from El Oro (H7, H8); and three from Loja (H4–H6) (see Additional file 1: Table S1). All these ITS2 sequences were overall similar to each other (see Table 2; Additional file 6: Alignment S2); there were no signs of pseudogene sequences in the chromatograms. The 720-bp *R. ecuadoriensis* 14-haplotype alignment (Additional file 6: Alignment S2) had 34 variable sites (4.7%), of which 18 were mutations (2.5%) and 16 were indels (2.2%). Within Ecuador, haplotypes H4 (Loja) and H13 (Manabí and Tsáchilas) differed by a single-nucleotide indel. Using the pairwise deletion option in MEGA X [61], we found an overall, uncorrected nucleotide diversity *π* = 0.0052 ± 0.0014 SE; the values were *π* = 0.0046 ± 0.0014 SE for Ecuadorian sequences and *π* = 0.0047 ± 0.0021 SE for Peruvian haplotypes, with a mean uncorrected between-group distance of 0.0062 ± 0.0019 SE. The ITS2-based K2p distance between the primarily palm-dwelling populations (Tsáchilas* vs* Manabí: 0.0031 ± 0.0012 SE) was somewhat smaller than the distances between these and the primarily nest-dwelling southern-Andean populations (0.0042 for Tsáchilas and 0.0049 for Manabí; both ±0.0014 SE). Table 2 shows K2p distances between population pairs; while overall low, ITS2-based distances were consistently larger in the comparisons involving Peruvian bugs.

For the ingroup + outgroup alignment (Additional file 7: Alignment S3; outgroup sequences deposited in GenBank under codes KT351069–KT351071), the smallest BIC model of nucleotide substitution included four rates (AC = GT; AG = CT; AT; and CG), a Gamma shape parameter (+*Γ*), and a proportion of invariable sites (+*I*). Phylogenetic analysis revealed no geographic or ecological structuring among Ecuadorian bugs, but the three ITS2 haplotypes from Peruvian specimens clustered in a separate clade with BPP = 1.0 (Fig. 7). This lent support to our *cytb* findings and, importantly, indicated that the similarity of mitochondrial DNA sequences across phenotypically and ecologically distinct Ecuadorian bugs (including the highly divergent Tsáchilas specimens; see also Fig. 8) is not due to introgression [78].

### Molecular analyses 3—species trees and Bayesian hypothesis testing

**Fig. 9:**
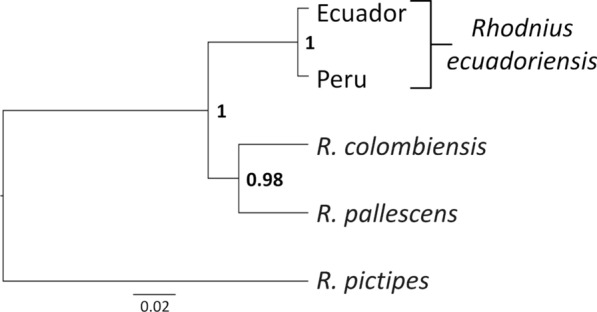
Multispecies coalescent analysis results: *Rhodnius ecuadoriensis* species tree estimated using mitochondrial *cytb* and nuclear ITS2 sequences under the two-lineage hypothesis (“Ecuador” and “Peru”). Maximum clade credibility tree based on 3000 replicate trees; Bayesian posterior probabilities for cladogenetic events are given close to each node. Scale bar is in substitutions/site. *Rhodnius colombiensis*, *R. pallescens* and *R. pictipes* were used as outgroup taxa

Table 3 summarizes the results of our assessment of the competing hypotheses about the number of independent lineages (one *vs* two) within *R. ecuadoriensis*. We found that the “two-lineage” hypothesis, H_1_, has very strong support from our DNA-sequence data, with log-mL estimates consistently larger (by > 24 and > 12 units, depending on the estimation procedure) than those of H_0_ (Table 3). These nested- and path-sampling BF estimates correspond to BPP between 0.999998 and 1.0 in favor of H_1_; conversely, then, we found that our data provide virtually no support for the “single-lineage” hypothesis (Table 3). Multispecies-coalescent analyses, therefore, substantiated locus-specific and size-free morphometric findings in that the best-supported species tree corresponds to the “two-lineage” hypothesis. This tree (Fig. 9) shows a well-supported *R. ecuadoriensis* clade within which our study specimens consistently segregate into two closely related lineages: (i) the Ecuadorian lineage, including typical southern-Andean bugs primarily from vertebrate tree-nests, atypical northern-Andean bugs primarily from wet-forest palms and intermediate northern-lowland bugs primarily from dry-forest palms; and (ii) the Peruvian lineage, including primarily nest-dwelling southern-Andean bugs (with naked-eye phenotypes similar to type material) from the Chicama and Mashcon-Marañón basins (Fig. 9; see also Figs. 1, 3, 4, 5, and 6).

**Table 3:** Marginal likelihoods, Bayes factors and hypothesis testing: one *versus* two independently evolving lineages in *Rhodnius ecuadoriensis*

Analyses and hypotheses	Pr(H)	Log-mL	SD	Log-BF	Pr(H|D)^a^
Nested sampling [69]					
H_0_: one lineage	0.5	− 3944.09	6.05	24.22	0
H_1_: two lineages (“Ecuador” and “Peru”)	0.5	− 3919.87	5.59	0	1
Path sampling^b^ [70]					
H_0_: one lineage	0.5	− 3888.23	–	12.94	< 0.00001
H_1_: two lineages (“Ecuador” and “Peru”)	0.5	− 3875.29	–	0	> 0.99999

*Pr*(*H*), Prior probability of each alternative hypothesis [here, both hypotheses are equally likely *a priori*: Pr(H_0_) = Pr(H_1_) = 0.5], *Log-mL* natural logarithm of the marginal likelihood, *SD* standard deviation of the log-mL, *Log-BF* natural logarithm of the Bayes factor (i.e. the difference in log-mL between H_1_ and H_0_), *Pr(H|D)* posterior probability of each hypothesis, given the data [here, Pr(H_0_|D) ≈ 0 and Pr(H_1_|D) ≈ 1 for both analyses]

^a^Estimated under the assumption of equal prior probabilities, as Pr(H_1_|D) ≈ BF/(1 + BF), and Pr(H_0_|D) = 1 − Pr(H_1_|D)

^b^Or “thermodynamic integration”; note that, in the implementation we used, this method does not provide SD estimates for the log-mLs

## Discussion

**Fig. 10:**
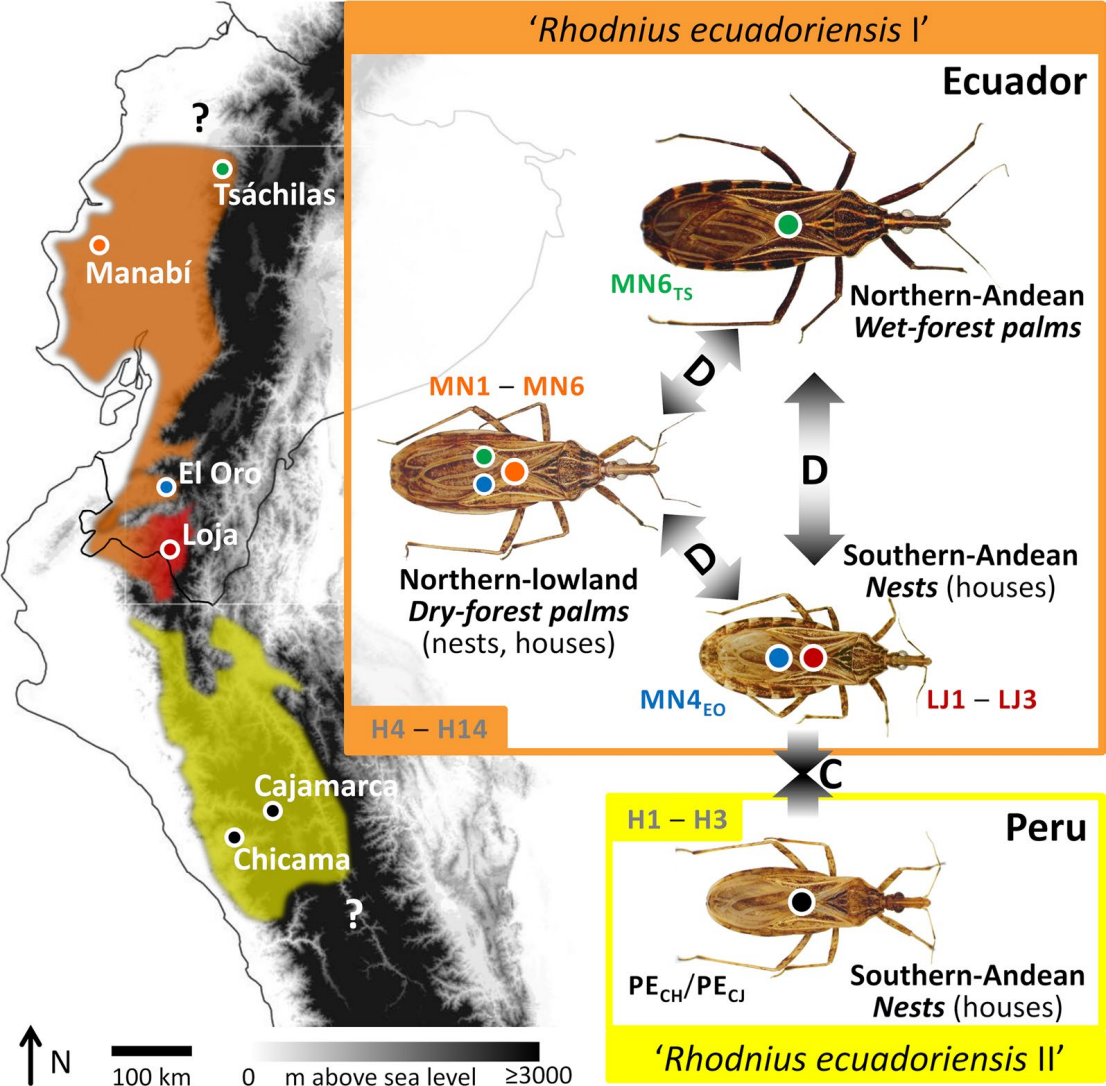
Divergence and convergence in Triatominae: genotypes, phenotypes and habitats of *Rhodnius ecuadoriensis* populations. The map illustrates the approximate distribution of the two major *R. ecuadoriensis* lineages: the Ecuadorian lineage (orange; “*Rhodnius ecuadoriensis* I” of [8, 18]) and the Peruvian lineage (yellow; “*Rhodnius ecuadoriensis* II” of [8, 18]). Question marks highlight uncertainties as to the species’ northern and southern range limits. The reddish shade in Loja suggests possible, partial differentiation of local populations in the Catamayo-Chira basin, as indicated by the identification of three closely related *cytb* haplotypes (LJ1–LJ3) not shared with other populations (Figs. 7, 8) and by limited microsatellite [41] and 2b-RAD (restriction site-associated DNA tag sequencing/genotyping based on type IIB restriction enzymes) genotyping data [79]. Colored circles show the approximate geographic location (on the map) of *cytb* haplotypes and their correspondence with each phenotype (on bug pictures); color codes are as in Figs. 1 and 5–8. Nuclear ITS2 haplotypes differ between Ecuadorian (H2–H14; orange box) and Peruvian bugs (H1–H3; yellow box), with no clear geographic, ecological or phenotype-related genetic structuring within Ecuador. The primary (natural) habitat of each population is given in bold italics. Gray-white arrows emphasize phenotypic divergence (*D*) or convergence (*C*) between populations

In this report we describe a striking instance of phenotypic divergence and convergence within a single nominal (i.e. named) species of Triatominae, *Rhodnius ecuadoriensis*. We found: (i) sharp, naked-eye phenotypic divergence of genetically similar Ecuadorian bugs (primarily nest-dwelling southern-Andean populations* vs* primarily palm-dwelling northern populations—and, within northern palm-dwelling bugs, Andean *vs* lowland populations); and (ii) marked, naked-eye phenotypic similarity, most likely due to convergence, of primarily nest-dwelling southern-Andean populations (northwestern Peru* vs* southwestern Ecuador) whose distinct DNA sequences and forewing (plus, to a lesser extent, head) shapes strongly suggest incipient evolutionary divergence (Fig. 10; Table 3; Additional file 4: Figure S1). Below we argue that local adaptation to distinct microhabitats is probably the key driver underpinning this remarkable example of phenotypic diversity within a single putative species.

Triatomines are blood-sucking bugs that live in sheltered microhabitats with a more-or-less stable food supply [1, 2, 27]. All *Rhodnius* species, for example, are primarily arboreal; most are tightly associated with palm-crown habitats, but some species and populations also exploit vertebrate nests built on tree branches, inside tree hollows, in bromeliads or on palm crowns [2, 27, 29]. Populations of a few *Rhodnius* species also occupy human-made habitats and can transmit *T. cruzi* to people and their domestic mammals [1, 3, 27]. *Rhodnius ecuadoriensis* is one such species. In the wild, it seems to be primarily associated with the endemic *Phytelephas aequatorialis* palm of western Ecuador, but has also been found in vertebrate nests; southern-Andean populations, in particular, occur in dry ecoregions in which palms are rare or absent, and appear to have shifted to squirrel, bird and opossum nests [1, 2, 26–34]. In addition, some *R. ecuadoriensis* populations can infest houses and peridomestic structures—with a preference for hen nests, guinea-pig pens and dovecotes [1, 24–27, 35, 36, 38, 39]. These synanthropic populations are major local vectors of human Chagas disease [1, 24–27, 36–39]; importantly, available phenotypic, genetic and behavioral evidence suggests that they most likely represent subsets of locally sympatric wild populations [36, 40–43]. At a broader spatial scale, *R. ecuadoriensis* is the only named *Rhodnius* species known to occur on the western side of the Andes south of the Magdalena-Urabá moist forests of northwestern Colombia; the Chocó rainforests along the Colombian Pacific coast separate *R. ecuadoriensis* from its sister-species clade, *R. pallescens*–*R. colombiensis* [8, 18]. Within its range, *R. ecuadoriensis* occurs in widely different ecoregions [8, 18, 25, 26]. In central-western Ecuador, presence records range from Andean wet premontane (or “cloud”) forests to semiarid parts of the coastal lowlands [25]. In southwestern Ecuador and northwestern Peru, the species occupies seasonally dry inter-Andean valleys up to 2700 m a.s.l. and is often found infesting houses [25, 26].

We reasoned that the broad ecological flexibility of *R. ecuadoriensis* was likely to correlate with similarly broad intraspecific variation, and set out to examine the signs of diversification and adaptation in this locally important vector species. To address both macro-scale diversity and micro-scale adaptations, we analyzed mitochondrial and nuclear DNA markers that have proven useful in similar study systems [5–9, 11, 12, 15–17, 19, 72–74, 80–84] and undertook a detailed qualitative/quantitative phenotypic assessment including head and forewing morphometrics [22, 40, 49, 51–53]; we then used rich, specimen-specific ecological metadata (Additional file 1: Table S1) to guide the interpretation of results.

### Macro-scale diversity: lineages and shape patterns

Our results provide strong support to the view that *R. ecuadoriensis* is composed of two major, independently evolving lineages [8, 18] (Table 3). The core Ecuadorian lineage has been dubbed “*R. ecuadoriensis* group I” [18]; it occupies highly diverse ecoregions from the wet central-western Ecuadorian Andes down to the drier valleys of the Catamayo-Chira basin—apparently always north of the Sechura desert-Huamaní range (Figs. 1, 10). The Peruvian lineage, or “*R. ecuadoriensis* group II” [18], occurs in the dry inter-Andean valleys of northwestern Peru, from the Huancabamba depression down to (and apparently excluding) the semiarid Santa river basin [8, 25, 26]; this distribution includes: (i) Pacific-slope valleys, from the eastern edge of the Sechura desert to the Chicama and perhaps Moche basins; and (ii) the Amazon-slope upper Marañón valley (Table 1; Figs. 1, 10). *Cytb* divergence levels suggest [17, 83] that these two lineages may have been evolving independently for 2.2–3.6 million years, with a late Pliocene–early Pleistocene most recent common ancestor. K2p distances (4.0–4.9%) are larger than those separating *R. prolixus* from its sister species, the partly sympatric *R. robustus* I (3.0–3.3%) [8, 17].

Although these differences are in the limit of what Wiemers and Fiedler [85] consider “low” levels of mitochondrial DNA K2p sequence divergence between reciprocally monophyletic sister clades, our ITS2 (Fig. 7) and multispecies coalescent results (Table 3; Fig. 9) lend further support to the hypothesis that Ecuadorian and Peruvian *R. ecuadoriensis* are independently evolving lineages [8, 18, 67]. An allozyme electrophoresis study [86] including *R. ecuadoriensis* colony bugs originally from Ecuador (Manabí and El Oro) and Peru (Cajamarca; same colony as PE_CJ_) provides additional insight into divergence at multiple nuclear loci; in particular, different alleles of *Mdh*, *Pep3* and *Pep4*, for which no heterozygotes were detected, segregated “according to the geographical origin of the specimens from Ecuador and Peru” ([86], p. 303). A later study showed that the 45S rDNA gene cluster occupies different chromosomal loci in Ecuadorian (X and Y chromosomes; bugs from Manabí) and Peruvian specimens (X chromosomes only; bugs from La Libertad) [87]. Taken together, our DNA results and these independent findings are strongly suggestive of a relatively long history of independent evolution of Ecuadorian and Peruvian *R. ecuadoriensis* lineages, likely involving ongoing or very recent speciation. Our detailed appraisal of phenotypes (Table 1; Figs. 3, 4; see also Additional file 3: Text S1) provides the basis for distinguishing bugs carrying Peruvian and Ecuadorian genotypes; we expect professional taxonomists to examine our findings and, if warranted, formally describe a new *Rhodnius* species based on Peruvian material.

Our DNA-based results, in addition, broadly mirrored those of forewing and (size-free) head shape analyses; as previously suggested [51, 53], explicit size partitioning was necessary to single out genetically distinct groups after traditional morphometrics. We expected wing shape to be relatively conserved because of the crucial role of flight in adult-bug dispersal [1, 27] and the importance of wing geometry for flight efficiency [88]. Forewing shape differences reflected the relatively deep genetic divergence of the Ecuadorian and Peruvian lineages (Figs. 6b, 7, 8). On the other hand, the adaptive value of head-shape variants within triatomine-bug species remains obscure. Our results suggest that, when size effects are removed, *R. ecuadoriensis* head shape may fairly mirror genetic divergence (Figs. 5e, 7, 8). In general, the elongated heads of northern, primarily palm-dwelling populations (Tsáchilas and Manabí) sharply contrast with the shorter, stouter heads of southern-Andean populations (Figs. 3, 5c, 6a). As discussed below, this may be related to a transition from palm to nest microhabitats [2, 89].

### Phenotypic variability at the micro-scale: microhabitat adaptations

Elongated heads and medium-sized bodies (relative to other triatomines) are characteristic of the genus *Rhodnius*, which mainly comprises palm-living species [1, 2, 29]. The few exceptions to this morphological “rule” seem to correspond to nest-dwelling species [2]. The *Psammolestes*, for example, are an atypical *Rhodnius* sub-lineage [5, 6, 8–10, 13] with strikingly distinct phenotypes—very small bodies and very short-and-stout heads [1, 2]. These clearly derived traits are probably related to the adaptation of the *Psammolestes* common ancestor to the enclosed vegetative nests of some ovenbirds [2, 18, 22]. Among *Rhodnius* species, the most similar in head shape and body size to the typical, southern forms of *R. ecuadoriensis* is *R. paraensis*, which has to date only been reported from the tree-hole nests of arboreal *Echimys* spiny rats [1, 2, 27, 90]. *Rhodnius domesticus* also has a relatively short and stout head for the genus; it is too among the few *Rhodnius* species not specializing in palm habitats—instead, it is associated with the nests and shelters of *Phillomys* tree-rats and *Didelphis* and *Marmosa* opossums in bromeliads and hollow tree-trunks [1, 2, 27].

These observations suggest that the reduced body size and head dimensions of typical *R. ecuadoriensis* populations may be a consequence of their shifting from the original palm-crown habitat to new, vertebrate-nest microhabitats [2]. This shift likely occurred in the dry Andean environments of southwestern Ecuador and northwestern Peru that overall lack native palm populations [25, 26, 30, 32, 39]. In Loja, wild *R. ecuadoriensis* often breed inside the nests of the tree-squirrel, *Sciurus stramineus/nebouxii* [2, 30, 32, 33, 91]. Within nest microhabitats, the close physical proximity between the (virtually ectoparasitic) bugs and their hosts would relax selection for long/narrow heads and mouthparts, which may be required for biting free-ranging hosts more safely (at a longer distance) and sucking their blood faster (thanks to larger cibarial-pump muscles) [89, 92]. In addition, and as has been also postulated for domestic triatomine populations [4], an overall more predictable food supply within a nest (or human dwelling), with a higher likelihood of repeated smaller blood meals, would relax the need for growing bigger bodies capable of storing larger amounts of blood [89]. Finally, host-mediated (passive) dispersal is probably more important among nest-dwelling than among palm-dwelling bugs, which might reduce the need for highly efficient flight, hence relaxing selection for elongated wings [27, 88]. We note that in Manabí *R. ecuadoriensis* occurs both in *Ph. aequatorialis* palms and in bird and mammal nests built on palms, trees or bromeliads [2, 28, 29, 31, 93]. This might help explain the intermediate phenotypes, including body size and head/forewing shape, of these primarily palm-dwelling lowland populations (Figs. 3, 5, 6; Additional file 4: Figure S1).

We also found a striking variability of overall color among *R. ecuadoriensis* populations. In particular, the dark hue of Tsáchilas bugs differs markedly from the typical brown-yellowish, straw-like color of the remaining populations (Fig. 3). This straw-like coloration is shared by *R. pallescens* and *R. colombiensis* [1, 77], suggesting that it is plesiomorphic (Additional file 4: Figure S1). In the fresh, field-caught bugs we studied, color variation involved mainly pigmentation intensity rather than discrete changes in the arrangement of markings. For example, some typically colored bugs may have a larger amount of irregular dark spots and markings, or their dark markings may have larger surfaces. The highly divergent Tsáchilas forms have large and abundant black markings on a reddish-brown, generally very dark background color. Although Tsáchilas and Manabí populations share *Ph. aequatorialis* as their primary ecotope, extensive field observations [28, 76] led us to notice that palm-crown microhabitats are often quite different in the wet Andes and the dry lowlands. In the dry Manabí lowlands, dead palm fronds and fibers tend to dry up, resulting in a straw-colored habitat substrate. In contrast, dead palm fronds and fibers quickly decay in the wet Andes foothills—where, in addition, large amounts of epiphytes grow on the palms. As a result, the palm-crown microhabitat of northern-Andean Tsáchilas bugs has a dark, actually reddish-brown, background color. Hence, the color of palm-dwelling bugs from each area (Fig. 3) closely matches their palm-microhabitat background, suggesting camouflage against the substrate [90, 94–96]. Also in line with this “camouflage hypothesis”, southern *R. ecuadoriensis* populations (Fig. 3) associate with rodent and/or bird nests made of light brown-yellowish materials—twigs, dry grass/leaves and straw. We therefore suggest that sight-guided predators likely provide the main selective pressure underlying color variability in *R. ecuadoriensis—*and probably in other triatomines.

### Caveats

The first, general caveat of this study is that the results are based on a relatively limited (albeit overall well-representative) sample of geographic–ecological populations and on only two genetic loci (albeit two that are informative for problems like the one we tackled). Our interpretations of these results, therefore, are best viewed as testable hypotheses to be addressed by future research. This should ideally include: (i) the study of natural populations along putative contact zones between genetic and phenotypic variants (e.g. on the Huamaní range or along the Andes foothills from Tsáchilas down to El Oro; Fig. 1), as well as in the Chocó wet forests of Ecuador and Colombia; (ii) population genetics/genomics analyses [41, 43, 79]; (iii) assessing the extent of range overlap and cross-fertility between lineages and populations [97]; or (iv) a detailed characterization of microhabitats, with an emphasis on bug/background color matching [98]. Second, the microhabitat associations we consider (Table 1) refer to the known primary habitats of natural wild populations [1, 2, 24–39]; we note, however, that the evidence of a primary link with vertebrate nests is still weak for wild Peruvian populations [2, 26, 27, 34]. Further, our southern-Andean samples came from human-made, not wild, microhabitats; as has been shown for southern-Andean Ecuadorian populations, we assume that the phenotypes [40] and genotypes [41] of these bugs do not differ significantly (or indeed at all) from those of their wild, nest-dwelling conspecifics (see also [36, 42]). This is also consistent with the patterns of genetic and/or morphometric similarity of wild and non-wild bugs described for northern-lowland *R. ecuadoriensis* [40, 43] and for other triatomine-bug species that often infest houses within their native ranges—including, for example, *R. prolixus* [82], *T. infestans* [52, 99], *T. brasiliensis* [100, 101] or *T. dimidiata* [102]. Finally, a small minority of the specimens we studied did not come from field collections. Seven bugs were from laboratory colonies (Additional file 1: Table S1), but, except for one head-size outlier (Fig. 5), we found no differences between these bugs and their field-caught relatives. Our sample also included two bugs from older collections (Additional file 1: Table S1); because the color of pinned bugs can change over time, we did not consider these two specimens in our qualitative assessment of phenotypes—for which we only used bugs that were fresh at the time of appraisal.

## Conclusions

Adaptation of an organism to its habitat becomes particularly evident when a human observer can predict habitat traits from organism traits. Our findings suggest that this is likely the case with *R. ecuadoriensis* populations. Thus, bug color predicts microhabitat background color, suggesting an adaptive response to selective pressure from sight-guided predators [90, 96]. The small body size and short/stout heads of southern-Andean bugs predict [2, 89] that wild populations preferentially exploit nest microhabitats—a proposition for which there is some empirical evidence, including abundant squirrel-nest populations [2, 30, 32, 33] and a strong association of domestic bugs with hen nests and guinea-pig pens [1, 26, 27, 35, 36, 39]. Importantly, we have also shown that populations with extremely divergent phenotypes can share their genetic backgrounds, at least for the two loci we examined; also importantly, our sequence data indicate that genetic similarity among Ecuadorian bugs is not due to mitochondrial DNA introgression [78]. In addition, our data reveal that southern-Andean *R. ecuadoriensis* populations with near-sibling naked-eye phenotypes belong in two distinct evolutionary lineages—the Ecuadorian “*R. ecuadoriensis* I” and the Peruvian “*R. ecuadoriensis* II” [18]. This can have implications for taxonomy and, hence, for the interpretation of taxonomy-dependent research results. We note, for example, that the reference *R. ecuadoriensis* strain kept at the LNIRTT (Fiocruz, Brazil) has the PE *cytb* haplotype, which is nearly 5% divergent from the LJ haplotypes that geographically correspond to the species’ type material from Catamayo, Loja [45]. In their classic revision, Lent and Wygodzinsky [1] illustrate *R. ecuadoriensis* with a Peruvian bug from Cajamarca (Fig. 257 in [1]). Peruvian- and Ecuadorian-lineage bugs are also clearly divergent in forewing and (size-free) head shape, at several allozyme loci [86] and cytogenetically [87].

In sum, our detailed appraisal of phenotypic and genetic diversity in *R. ecuadoriensis* revealed phenotypic divergence within genetically homogeneous populations and phenotypic convergence of genetically distinct lineages likely on their way to speciation—if not separate species already. Such remarkable, bidirectional phenotypic change within a single nominal taxon was apparently associated with adaptation to particular microhabitats. These findings shed new light on the origins of phenotypic diversity in the Triatominae, warn against excess reliance on phenotype-based triatomine-bug systematics, and confirm the Triatominae as an informative model-system for the study of phenotypic change under ecological pressure.

## Supplementary Information

**Additional file 1: Table S1.** Populations, specimen details and haplotype codes of 106 *Rhodnius ecuadoriensis* bugs used in morphometric and/or molecular analyses. A summary table with the numbers of bugs used in each analysis is also provided.

**Additional file 2: Alignment S1.** Mitochondrial cytochrome *b* haplotypes in *Rhodnius ecuadoriensis* from Ecuador and Peru, plus outgroup species (*R. colombiensis*, *R. pallescens*, *R. pictipes*).

**Additional file 3: Text S1.** Detailed descriptions of the diverse *Rhodnius ecuadoriensis* phenotypes.

**Additional file 4: Figure S1.** Phenotype–microhabitat–phylogeny correspondences. Multispecies coalescent species tree (as in Fig. 9 of the main text), with pictures (approximately to the same scale) of adult *Rhodnius ecuadoriensis* and its closest relatives—*R. colombiensis*, *R. pallescens* and *R. pictipes*. The distribution of phenotypes along the phylogeny suggests that the common ancestor of the diverse *R. ecuadoriensis* forms was most likely a relatively large, straw-like-colored bug. Similarly, the distribution of primary microhabitats suggests that a shift of southern-Andean populations from palm crowns (green stars) to vertebrate nests (orange circles) resulted in convergence towards the small-size, short-head/wing typical *R. ecuadoriensis* phenotype; the combined star/circle symbol indicates that northern-lowland Manabí bugs are primarily palm-dwelling but may also exploit nest microhabitats. *Rhodnius ecuadoriensis* populations:* N-A* Northern-Andean (Tsáchilas),* N-L* northern-lowland (Manabí),* S-A* southern-Andean (El Oro and Loja in Ecuador; La Libertad and Cajamarca in Peru).

**Additional file 5: Figure S2.** Centroid-size comparisons. Population boxplots and Tukey-Kramer (T-K) tests for head and forewing centroid sizes derived from geometric morphometrics.

**Additional file 6: Alignment S2.** Fourteen nuclear ITS2 haplotypes found in *Rhodnius ecuadoriensis* from Ecuador and Peru.

**Additional file 7: Alignment S3.** Nuclear ITS2 haplotypes in *Rhodnius ecuadoriensis* from Ecuador and Peru, plus outgroup species (*R. colombiensis*, *R. pallescens* and *R. pictipes*).

## Data Availability

Data supporting the conclusions of this article are included in the article and its additional files. DNA sequences have been deposited in GenBank under accession numbers MT497021–MT497038 for mitochondrial *cytb* haplotypes and KT267937–KT267950 plus KT351069–KT351071 for nuclear rDNA ITS2 haplotypes.

## Abbreviations

BF: Bayes factor
BIC: Bayesian information criterion
BPP: Bayesian posterior probability
CV: Canonical variate
CVA: Canonical variate analysis
*cytb*: Mitochondrial cytochrome* b* gene
EO: El Oro province, Ecuador
ESS: Effective sample size
ITS2: Nuclear ribosomal second internal transcribed spacer
K2p: Kimura two-parameter genetic distance
LJ: Loja province, Ecuador
LNIRTT: Laboratório Nacional e Internacional de Referência em Taxonomia de Triatomíneos, Fiocruz, Brazil
MCMC: Markov chain Monte Carlo
mL: Marginal likelihood
MN: Manabí province, Ecuador
PC: Principal component
PCA: Principal component analysis
PE: Peru
SE: Standard error
TPS: Thin plate spline
TS: Santo Domingo de los Tsáchilas province, Ecuador
2b-RAD: Restriction site-associated DNA tag sequencing/genotyping based on type IIB restriction enzymes
