# Correction to: Under pressure: phenotypic divergence and convergence associated with microhabitat adaptations in Triatominae

**DOI:** 10.1186/s13071-021-04729-y

**Published:** 2021-05-03

**Authors:** Fernando Abad-Franch, Fernando A. Monteiro, Márcio G. Pavan, James S. Patterson, M. Dolores Bargues, M. Ángeles Zuriaga, Marcelo Aguilar, Charles B. Beard, Santiago Mas-Coma, Michael A. Miles

**Affiliations:** 1 Núcleo de Medicina Tropical, Faculdade de Medicina, Universidade de Brasília, Brasília, Brazil; 2 Faculty of Infectious and Tropical Diseases, London School of Hygiene and Tropical Medicine, London, UK; 3 Laboratório de Epidemiologia e Sistemática Molecular, Instituto Oswaldo Cruz-Fiocruz, Rio de Janeiro, Brazil; 4 Division of Vector Borne Diseases, Centers for Disease Control and Prevention, Fort Collins, USA; 5 Laboratório de Mosquitos Transmissores de Hemato-zoários, Instituto Oswaldo Cruz-Fiocruz, Rio de Janeiro, Brazil; 6 Departamento de Parasitología, Facultad de Farmacia, Universidad de Valencia, Valencia, Spain; 7 Facultad de Ciencias Médicas, Universidad Central del Ecuador, Quito, Ecuador; 8 Instituto Juan César García, Quito, Ecuador

## Correction to: Parasites Vectors (2021) 14:195 https://doi.org/10.1186/s13071-021-04647-z

Following publication of the original article [1], it was brought to our attention that the second author had (erroneously) not been marked as the second corresponding author.

The original article has since been corrected and the corrected corresponding author information may be found in this correction.

The authors apologize for any inconvenience caused.
