# Supplementary material for: Under pressure: phenotypic divergence and convergence associated with microhabitat adaptations in Triatominae
Source: Parasit Vectors. 2021 Apr 8;14:195. doi: 10.1186/s13071-021-04647-z (PMC8034103; doi:10.1186/s13071-021-04647-z)
Supplement: Supplementary file 3 — Additional file 3: Text S1. Detailed descriptions of the diverse Rhodnius ecuadoriensis phenotypes. [file 13071_2021_4647_MOESM3_ESM.pdf]

S1 Text. Detailed descriptions of the diverse *Rhodnius ecuadoriensis* phenotypes.

(Adult bugs; Figure numbers refer to the main text.)

Southern-Andean 1: southwestern Ecuadorian (El Oro-Loja) typical forms

Bugs collected in/around houses in the southwestern Ecuadorian provinces of El Oro (Puyango basin) and Loja (Catamayo-Chira basin) were virtually identical to the type material (Figs 1 and 3). These are very small triatomines, with total lengths of 12.50–13.50 mm in males and ~14.50 mm in females. Adult bugs are light brown-yellowish with dark brown stripes and irregular markings on the body and appendages, a pattern that is especially conspicuous on the legs. The head is granulose, relatively short (2.39 mm, range 2.17–2.55 in El Oro; 2.30 mm, range 2.09–2.51 in Loja) and stout for the genus, approximately twice as long as wide across the eyes (length:width ratio 1:0.49–0.56 in both populations; Fig 5), and very slightly longer than the pronotum. Eyes are medium-sized (measurement E in Fig 2, ~0.70–0.90 mm). The second segment of the antennae is light-brown basally and darker apically, whereas the third segment is basally dark and apically lighter. The second rostral segment does not attain the neck, but reaches or slightly surpasses the level of the ocelli. The neck is dark on the sides, with a light, broad medial dorsal band. The anterior lobe of the pronotum is granulose, with anterolateral angles not very prominent; the posterior lobe is rugose-granulose and light brown with darker mottling. Submedian carinae and lateral margins are yellowish; the spaces between them have a mottled pattern with dark spots forming two dark, poorly defined stripes in each of these spaces. The scutellum has 2+2 well-defined, yellowish anterior carinae that fuse into 1+1 on the body of the scutellum and then into a single median carina on the posterior process, whose upper surface is light-colored. The hemelytra are straw-colored, including veins; membranes have an overall mottled

aspect, with irregular dark stripes and spots and lighter dots. Legs are light yellowish brown with a conspicuous mottled pattern; irregular dark brown stripes and spots cover the surface of femora (where the pattern is more conspicuous and dark markings concentrate on the distal third) and tibiae (with darker distal extremes). The ventral surface of abdomen is light brown-yellowish, irregularly mottled with dark brown. Dorsally, each segment of the connexivum has a dark brown spot covering the anterior 1/3 to 1/2. The median process of the pygophore is shortly triangular and has a pointed apex (S1 Fig).

Southern-Andean 2: northwestern Peruvian (Chicama-Cajamarca) typical forms

Peruvian *R. ecuadoriensis* bugs made available by Prof CA Cuba Cuba were field-collected in human environments in the dry middle-upper Chicama basin (Cascas district, department of La Libertad, ~350 km south of our southernmost Ecuadorian fieldwork sites), except for one specimen collected in Suyo (department of Piura, near the border with Loja in the Catamayo-Chira basin) (Figs 1 and 9). The overall aspect of these bugs matches that of the type material described above; however, while the specimen from Suyo could not be distinguished from those collected in Loja or El Oro, Chicama bugs were noticeably lighter than Ecuadorian specimens (Fig 3). This was more evident on the legs – in Peruvian bugs, the dark mottled pattern was limited to small clusters of dots and stripes on the basal and distal thirds of femora (leaving a central, broad yellowish area with few or no black markings) and tibiae (Fig 3). The posterior lobe of the pronotum was also overall lighter than in Ecuadorian material, and the anterolateral angles more rounded and less prominent (Fig 3). Peruvian bugs were also more slender than the typical specimens from El Oro-Loja; their legs, for example, tended to be longer and thinner. Width:length ratios for hind and medial femora were

1:8.82–9.20 and 1:7.20, respectively (Chicama), *vs.* 1:8.81 and 1:5.70 (typical specimens from Loja). The pronotum was also noticeably wider in typical material than in Peruvian bugs, with length:width ratios of 1:1.43 (Peru) *vs.* 1:1.68 (Loja). The heads of Peruvian bugs were also relatively short and stout; on average, they were ~2.55 mm long (range, 2.40–2.68 mm) and ~1.26 mm wide (1.17–1.32 mm), for a length:width ratio of 1:0.47–0.52 (Fig 5).

Northern-lowland: Manabí dry-forest palm forms

Phytelephas aequatorialis palms often harbor *R. ecuadoriensis* populations in the central coastal province of Manabí, Ecuador. The overall aspect and coloration of these bugs largely match those of typical *R. ecuadoriensis*, but Manabí bugs are noticeably larger than the type material (Figs 3, 9, and S2 Fig): total length is ~16.00 mm in females and ~14.50–15.00 mm in males. The heads are also longer than in domestic specimens, with average values typically >2.95 mm (range, 2.60–3.20 mm); the length:width ratio is usually 1:0.40–0.47 (Fig 5). Legs are longer and more slender than in typical specimens, with hind-leg ratios of 1:1.40 (femur:tibia length) and 1:11.50 (femur width:length). Light-colored specimens are somewhat similar to Peruvian material in that the irregular dark markings tend to concentrate on the basal and distal thirds of the femora (Fig 3).

Northern-Andean: Tsáchilas wet premontane-forest palm forms

In 1998, a male *Rhodnius* specimen was collected near Alluriquín, by the road Quito-Santo Domingo (province of Pichincha, currently Santo Domingo de los Tsáchilas), on the western Andes foothills at ~900 m above sea level, and brought to us for identification. The site is within the range of *R. ecuadoriensis*, which is not shared by any other known *Rhodnius* species, but the overall morphology and coloration of the

specimen differed strikingly from those of the type material (Figs 3, 4, 9, S1 Fig, and S2 Fig). The bug was noticeably larger and remarkably darker than reported for *R. ecuadoriensis*, but smaller than the closely-related species *R. pallescens* and *R. colombiensis*. The head of this specimen was much longer than in typical *R. ecuadoriensis* (Fig 4), but shorter than in *R. pallescens* (≥4.5 mm) or *R. colombiensis* (~4 mm). Field surveys were carried out in the area of Andean wet montane forests where this specimen was collected (Fig 1), and abundant material was recovered from *Ph. aequatorialis* palms using live-baited adhesive traps. The following detailed description is based on that material.

Small triatomine (body length of males 17.00 mm, of females 17.70 mm); overall color dark brown, blackish in many live specimens, with lighter brown markings (with an orange-reddish dye in many specimens) on various parts of the body and appendages (Figs 3, S1 Fig, and S2 Fig). Irregularly shaped dark spots are present on legs, but they are inconspicuous on the slightly lighter brown-reddish background. A short golden pilosity is present on most of the integument. Head granulose (more conspicuously on lateral view, especially in the post-ocular region; Fig 4), 3.50 mm in length (range 3.20–3.70 mm), almost 2.5 times as long as wide across the eyes (length:width ratio 1:0.39–0.43; Fig 5), and slightly longer than the pronotum. Short golden setae emerge from head granules, many of which are orange-brownish on an overall black background. The head is indeed dark brown-blackish, except for these granules and a narrow dorsal, median light brown-reddish stripe that covers the longitudinal median prominence (Figs 3 and 4). 1+1 light longitudinal light brown-orange stripes are also present in the postocular region under ocelli; the medium-sized eyes surpass the level of the lower surface but do not attain that of the upper surface of the head (Fig 4). First segment of

antennae not attaining the apex of the head, and covered by scarce short hairs; second segment entirely dark brown (slightly lighter at its base in some fair individuals), covered by relatively abundant short pilosity and with scarce, long and slender trichobothriae; third segment light brown yellowish distally but dark on the basal 1/4th, with abundant short adherent hairs and longer, evenly distributed trichobothriae; fourth segment pale yellowish with dark apex and pilosity similar to that of the third segment. The first segment of the rostrum reaches the site of emergence of antennae on the antenniferous tubercle; the second segment is long, attaining the neck; the third segment reaches the inferior groove of the prosternum in which the stridulatory sulcus is located, attaining the acetabuli of fore coxae. Pilosity scarce on the rostrum: short setae on first and second segment, with longer and more slender, straight setae on the third segment. The neck is dark brown-blackish, with light brown-reddish longitudinal stripes on the median area of dorsal side and below the level of ocelli (Figs 3 and 4).

Pronotum lobes separated by a pronounced transversal groove; the submedian carinae are conspicuously curved as they cross this groove (Fig 3). Anterior lobe granulose, with a very dark, brown-blackish background and a few small tubercles (with very short setae) in the spaces between submedian carinae and lateral margins. The space between carinae is black (an X-like shape is visible in some lighter bugs) and divided by a marked, median longitudinal furrow. Anterolateral angles not very prominent but slightly pointed, black with external margins and tips light brown yellowish. Posterior lobe rugose-granulose, dark brown blackish with lighter brown tubercles regularly distributed in the spaces between carinae and between lateral margins and carinae; this arrangement results in an overall spotted pattern. Carinae and pronotum margins light brown (reddish in many specimens), contrasting against the

dark background. Posterolateral angles not very prominent (Fig 3). The scutellum is rugose, dark brown to black with 2+2 yellowish basal carinae that merge in 1+1 carinae at the body of the scutellum and then into a single carina, with light color extending to the posterior process. The central furrow between submedian carinae has 6–7 marked, rough transversal rugosities. The posterior process is almost as long as the body of the scutellum, yellowish on its upper side as a continuation of median carina. It is rugose on its base and has a rounded tip, lighter than the base (Fig 3).

Overall aspect of legs dark brown, with short pilosity on their entire surface; irregularly shaped blackish markings overlay a slightly lighter, reddish-brown background. This mottled pattern is more evident on coxae and throchantera (where dark markings are very conspicuous and background color is manifestly reddish in most specimens) and on femora (Fig 3), but hardly noticeable on tibiae, whose distal third is almost black and covered by dense pilosity. Narrow, longitudinal black stripes are present on femora, and are particularly noticeable on the hind pair of legs. Legs are long and slender (femora width:length ratio 1:6.90–9.60; femora:tibiae length ratio 1:1.18–1.45). The distal denticle of the femora is much more developed than in other populations, particularly in the fore legs (Fig 3G inset). Tarsi are covered by long hairs, with spongy fossulae in the fore and medium pairs of legs of both sexes.

Hemelytra brown, not attaining the hind tip of the abdomen; membranes are slightly rugose and brown with round, lighter, confluent spots. Venations light brown, conspicuous against the darker background. Corium well differentiated from membranes, with lighter venations and darker cells; the marginal cell has a lighter central area (Fig 3). Abdomen dark brown, covered by pale short setae and with an overall pattern of light and dark spots (Fig 3). Urosternites are blackish brown with a

lighter spotted pattern and an arrow-shaped, median lighter marking; spiracles are visible as light round spots on the darker part of urosternites close to the margins (Fig 3). The integument of urosternites is finely granulose and transversally striate. Connexival segments dark brown-blackish. Yellowish markings (with reddish dye in many specimens) cover the posterior fourth of urotergites, including the suture and projecting forwards on the external half of segments, reaching the posterior 1/3rd of the marginal half of each tergite (Fig 3 and S1 Fig). The median process of the pygophore is triangular; its apex is not evidently pointed – instead, the tip is truncated in most of the bugs we examined (S1 Fig).
