# Supplementary figures and images for: Under pressure: phenotypic divergence and convergence associated with microhabitat adaptations in Triatominae
Source: Parasit Vectors. 2021 Apr 8;14:195. doi: 10.1186/s13071-021-04647-z (PMC8034103; doi:10.1186/s13071-021-04647-z)

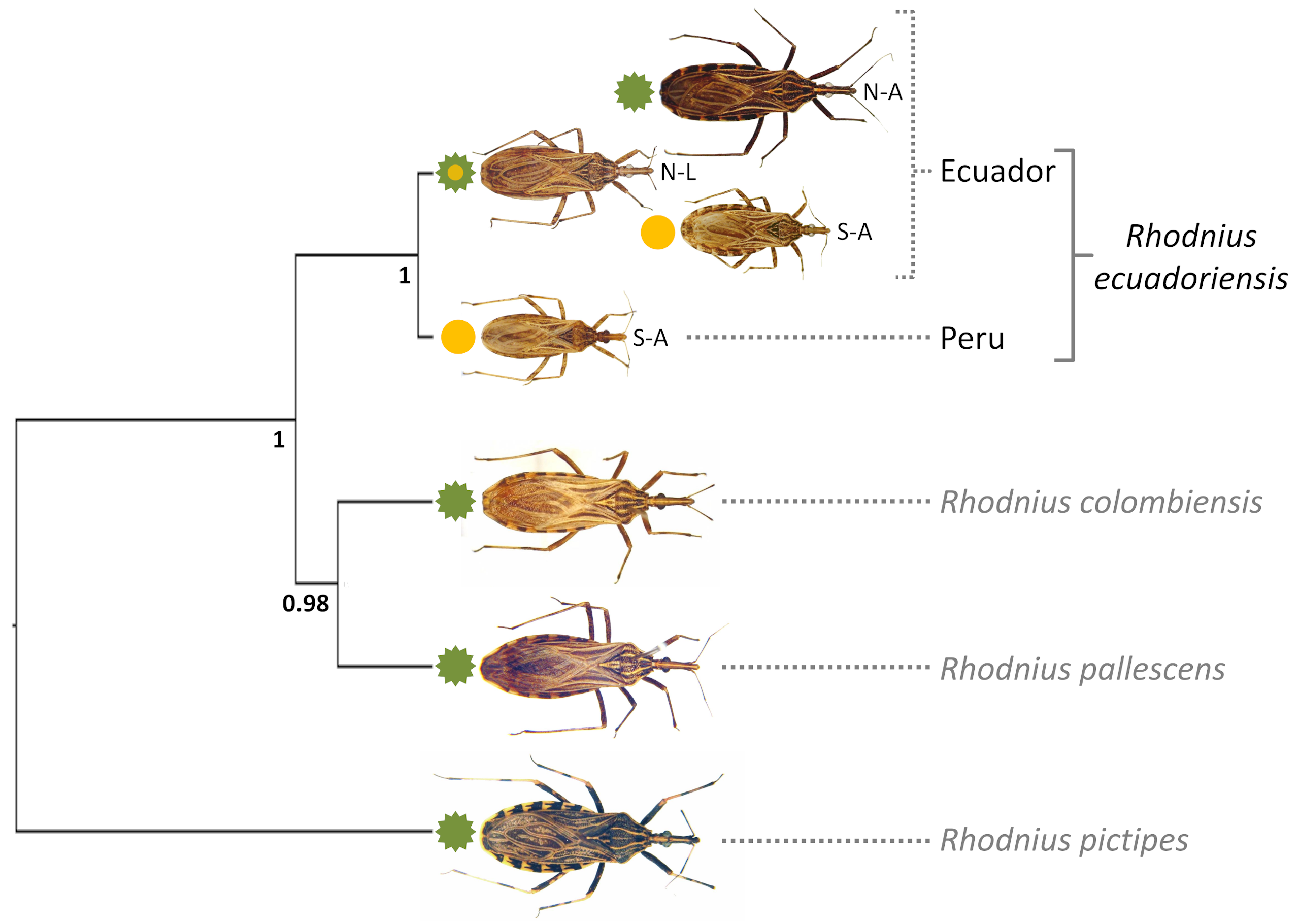

Supplement: Supplementary file 4 — Additional file 4: Figure S1. Phenotype–microhabitat–phylogeny correspondences. Multispecies coalescent species tree (as in Fig. 9 of the main text), with pictures (approximately to the same scale) of adult Rhodnius ecuadoriensis and its closest relatives—R. colombiensis, R. pallescens and R. pictipes. The distribution of phenotypes along the phylogeny suggests that the common ancestor of the diverse R. ecuadoriensis forms was most likely a relatively large, straw-like-colored bug. Similarly, the distribution of primary microhabitats suggests that a shift of southern-Andean populations from palm crowns (green stars) to vertebrate nests (orange circles) resulted in convergence towards the small-size, short-head/wing typical R. ecuadoriensis phenotype; the combined star/circle symbol indicates that northern-lowland Manabí bugs are primarily palm-dwelling but may also exploit nest microhabitats. Rhodnius ecuadoriensis populations: N-A Northern-Andean (Tsáchilas), N-L northern-lowland (Manabí), S-A southern-Andean (El Oro and Loja in Ecuador; La Libertad and Cajamarca in Peru). [file 13071_2021_4647_MOESM4_ESM.tif]

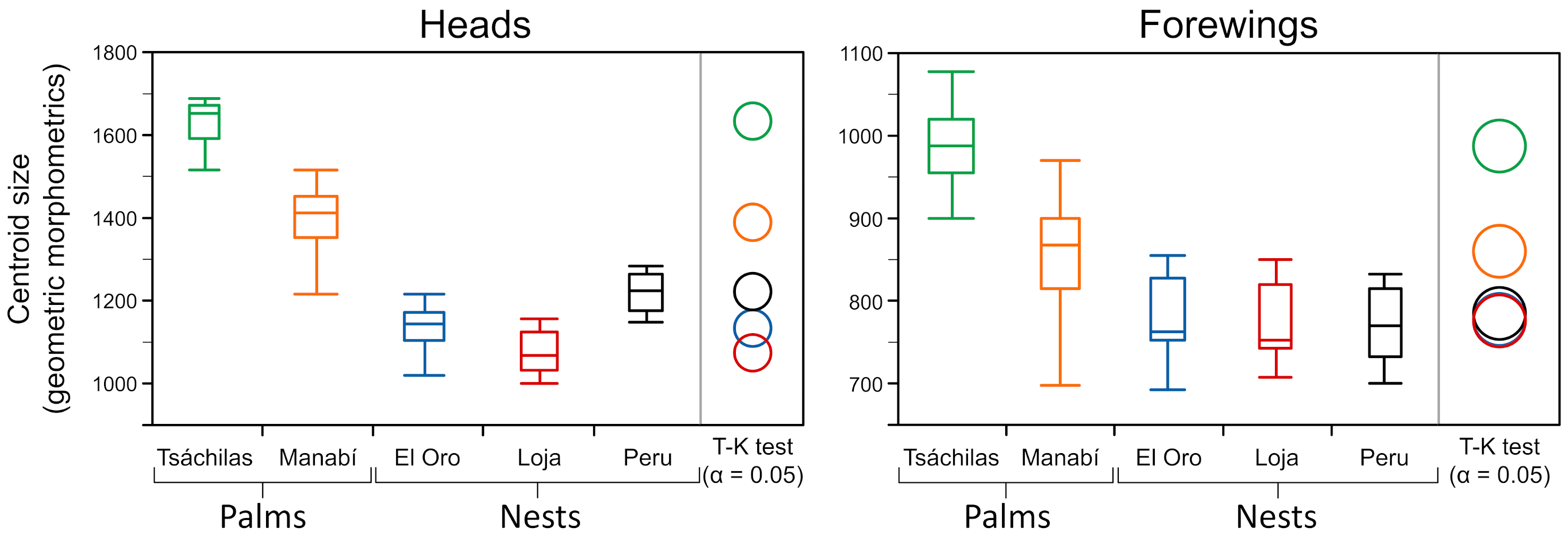

Supplement: Supplementary file 5 — Additional file 5: Figure S2. Centroid-size comparisons. Population boxplots and Tukey-Kramer (T-K) tests for head and forewing centroid sizes derived from geometric morphometrics. [file 13071_2021_4647_MOESM5_ESM.tif]
